# HMGA2 Stabilizes hnRNPK to Drive YAP-Dependent Liver Metastasis in Pancreatic Cancer

**DOI:** 10.7150/ijbs.133139

**Published:** 2026-10-02

**Authors:** Yu Xie, Hexing Hang, Haobai Liu, Nannan Wang, Chengzhi Wang, Yifei Yang, Lei Fang, Yudong Qiu, Hao Cheng

**Affiliations:** 1 Department of Pancreatic and Metabolic Surgery, Nanjing Drum Tower Hospital, Affiliated Hospital of Medical School, Nanjing University, Nanjing, China.; 2 State Key Laboratory of Pharmaceutical Biotechnology, Jiangsu Key Laboratory of Molecular Medicine, Chemistry and Biomedicine Innovation Center, Medical School of Nanjing University, Nanjing, China.

**Keywords:** HMGA2, pancreatic cancer, liver metastasis, hnRNPK, YAP

## Abstract

Pancreatic cancer (PC) is a highly aggressive malignant neoplasm with a propensity for liver metastasis (LM), which significantly contributes to poor patient prognosis, but its molecular mechanism remains incompletely elucidated. In this study, we demonstrated that high mobility group protein A2 (HMGA2) is markedly elevated in LM tissues and circulating tumor cells (CTCs) of PC patients. Mechanistically, HMGA2 binds to heterogeneous nuclear ribonucleoprotein K (hnRNPK) via “AT-hook” functional motif, suppressing its ubiquitination to block proteasomal degradation. This interaction promotes the transcriptional activation of Yes-associated protein (YAP) by facilitating hnRNPK binding to the YAP promoter and thereby enhancing metastatic potential of PC. Functional assays validate that hnRNPK mediates HMGA2's pro-metastatic function, and YAP acts as the indispensable downstream effector of this cascade. Our findings establish the HMGA2/hnRNPK-YAP axis as a critical regulator of LM and propose targeting this pathway as a promising therapeutic strategy for metastatic PC.

## Introduction

Pancreatic cancer (PC) constitutes a highly malignant neoplasm originating from the digestive system 1. Data from the Cancer Surveillance Center shows that PC ranks third among cancer-related deaths, and it is estimated that it may rise to 2nd place by 2030 2. PC progresses rapidly with insidious and atypical early symptoms, leading most patients to be diagnosed at an advanced stage or accompanied by distant metastasis, wherein the liver emerges as the most prevalent metastatic destination 3. Despite recent advances in the diagnosis and management of PC, liver metastasis (LM) remains a leading cause of mortality among patients 4. Surgical resection is currently the only effective means to provide PC patients with a chance of cure and long-term survival 5. However, more than 80% of patients are unable to undergo surgical treatment due to advanced disease or LM 6. Even after surgical resection, more than 40% of patients still develop LM within 3 years 7. Therefore, an in-depth study of the molecular mechanisms of LM is of great importance for guiding patients' clinical decision-making and improving the prognosis of PC patients.

As a non-histone chromatin-binding protein, high mobility group protein A2 (HMGA2) contributes critically to the maintenance and modulation of DNA function, including participation in processes such as replication, recombination, transcription and repair as an important member of the HMG family of proteins 8. HMGA2 exhibits widespread expression in undifferentiated cells during the early phases of embryogenesis whereas its expression becomes more restricted in late development and adulthood, but it is re-expressed in a variety of tumor tissues 9. Recent investigations have revealed elevated HMGA2 expression in diverse malignancies, encompassing lung, gastric and breast cancers 10. Furthermore, it promotes tumorigenesis through a variety of mechanisms and is involved in almost all aspects of cellular events, including cell proliferation, apoptosis, senescence, metastasis, DNA repair and stem cell self-renewal 11. It has also been shown that HMGA2 is involved in epithelial-mesenchymal transition (EMT) and associated with numerous signaling pathways exemplified by Wnt/β-catenin and TGF-β/Smad 12. Chen et al. found that the formation of circNSUN2/IGF2BP2/HMGA2 ternary complex reinforces the stability of HMGA2 mRNA and thus promotes the LM progression of colorectal cancer 13. In addition, studies have indicated that elevated HMGA2 expression correlates closely with PC progression and chemoresistance, and it has been established as a marker of basal-like disease in PC 14-16. However, the role of HMGA2 in LM of PC and its functional regulatory features remain insufficiently investigated.

In this study, we demonstrated that HMGA2 promoted LM progression of PC by binding with heterogeneous nuclear ribonucleoprotein K (hnRNPK) and then activating the transcription of Yes-associated protein (YAP). Thus, targeting HMGA2/hnRNPK-YAP axis may provide a potentially feasible therapeutic strategy for LM of PC.

## Materials and Methods

### Clinical samples

For RNA-seq, treatment-naive patients with suspected PC and oligometastatic liver disease (≤3 metastatic lesions confined to the liver without extrahepatic dissemination 17) determined by preoperative imaging and multidisciplinary team evaluation were enrolled. Following laparoscopic confirmation of oligometastatic liver disease, primary PC and matched LM tissues were collected via laparoscopic biopsy synchronously (both tissue types procured during a single laparoscopic procedure) for definitive diagnosis. All patients were deemed unresectable according to the National Comprehensive Cancer Network (NCCN) guidelines and had peripheral blood samples collected prior to treatment. The diagnosis of LM of PC was finally based on histopathologic examination. For Western blot, PC and matched normal pancreatic tissues were obtained from radical pancreatectomy. No specimens were obtained from separate percutaneous palliative biopsy or autopsy cases in this cohort. All patients enrolled in this study furnished written informed consent, and full clinicopathological characteristics of all involved patient cohorts are provided in Table S1-3. The human PC tissue microarray (PanSur2201) was purchased from AiFang Biological (Nanjing, China).

### RNA-seq

Library construction and sequencing services were commissioned to Genechem (Shanghai, China). Briefly, poly(A) selection was used for mRNA enrichment; after fragmentation, mRNA was reverse-transcribed into cDNA. After completing library preparation, PE150 sequencing was conducted on the Illumina NovaSeq 6000 platform (≥6 Gb data per sample). Raw sequencing reads were subjected to quality control using FastQC, trimmed via Trimmomatic, and quantified with featureCounts or StringTie. DESeq2 was used for differential gene expression profiling, filtered by |log₂FC| ≥ 1.5 and adjusted P < 0.05.

### Quantitative Real-time polymerase chain reaction (RT-qPCR)

Trizol reagent (15596026CN, Thermo Fisher) was used to extract total RNA, and then qRT SuperMix (R222-01, Vazyme) was used to synthesize cDNAs. ChamQ Universal SYBR qPCR Master Mix (Q711-02, Vazyme) was used to conduct RT‒qPCR. Gene expression was normalized to β-actin and quantified via the 2^-△△Ct^ method for relative analysis. The primer sequences were as follows: HMGA2-F: GCAGCAGCAAGAACCAACCG, HMGA2-R: CTTCCCAGGCAAGGCAACAT; ACTIN-F: CATGTACGTTGCTATCCAGGC, ACTIN-R: CTCCTTAATGTCACGCACGAT.

### Western blot analysis

Cells were subjected to lysis with ice-cold RIPA buffer (AR0105, Beyotime), followed by a 10-minute ice incubation. The resulting protein lysates were subjected to 10% SDS-PAGE, followed by electroblotting transfer onto methanol-activated PVDF membranes. The membranes were then incubated overnight at 4°C with a target-specific primary antibody. The antibodies utilized are specified as follows: anti-HMGA2 (#8179, CST); anti-β-actin (AC026, ABclonal); anti-Vimentin (A2584, ABclonal); anti-E-cadherin (20874-1-AP, Proteintech); anti-N-cadherin (#13116, CST); anti-hnRNPK (A0772, ABclonal), anti-YAP (A19134, ABclonal) and anti-Ubiquitin (A19686, ABclonal).

### Circulating tumor cells (CTCs) enrichment

CytoploRare Kit (Geno Biotech, Shanghai, China) was used to enrich and enumerate CTCs. Peripheral blood (3 mL) was collected into EDTA tubes, lysed with RBC lysis buffer (4°C, 15 min), centrifuged at 300×g for 5 minutes, and the pellet was washed twice. The sample was incubated with anti-CD45/CD14 magnetic beads (4°C, 1 h), magnetically separated, and washed. Enriched cells were incubated with anti-CK and anti-FRα antibodies to be detected. According to the manufacturer's specifications, this negative enrichment method achieves a mean CTCs recovery rate of 90.0% and leukocyte removal efficiency >95%.

### Immunofluorescence (IF)

At room temperature, cells were first immobilized with 4% paraformaldehyde for 15 minutes, then rendered permeable with 0.1% Triton X-100 for 10 minutes, and non-specific binding sites were blocked with 5% BSA for 1 hour. After primary antibodies were incubated with cells overnight at 4°C, Alexa Fluor-conjugated secondary antibodies were applied for 1 hour. DAPI was used for nuclear counterstaining, and confocal microscopy was employed for image acquisition.

### Single-cell RNA-seq analysis

The public single-cell RNA-seq dataset GSE197177 and GSE212966 containing a total of 4 paratumor, 8 primary pancreatic tumor and 4 liver metastatic tissues were reanalyzed. Raw counts were processed with R package Seurat (v5.3.0), with cell filtering criteria set as follows: nFeature_RNA between 300 and 7000, mitochondrial gene percentage <10%, hemoglobin-related gene percentage <3% and nCount_RNA >1000. Cell types were annotated by canonical markers: T/NK cells (CD3D), macrophage (CD68), fibroblast (COL1A2), epithelial (EPCAM), B cells (MS4A1), endothelial cells (CDH5), stellate cells (MYH11), mast cells (TPSAB1), neutrophil (FCGR3B), plasma cells (IGHG1), MKI67+ cells (MKI67) and endocrine cells (SST). After cell annotation, the epithelial subpopulations were extracted and re-clustered, yielding four distinct subpopulations. Malignant epithelial cells were identified using CytoTRACE (v0.3.3) and inferCNV (v1.24.0). For the 24 PDAC and 11 normal pancreas comparison, gene expression data were retrieved from the scCancerExplorer online database (https://bianlab.cn/scCancerExplorer/home).

### Cell lines and culture

HEK293T and Human PC cell lines were purchased from the Procell (Wuhan, China); Mouse PC cell line KPC and Panc02 were kindly provided by Dr. Shu Zhang (Nanjing Drum Tower Hospital, Nanjing, China). The KPC cell line was isolated from KPC mice (*LSLKras^G12D^/LSLTrp53^R172H/+^/Pdx1-Cre*) of KRAS-driven murine pancreatic ductal adenocarcinoma. Cells were maintained in DMEM (Wisent, Canada) added with 10 % FBS (CG0430B, Celligent) at 37 ℃ in a 5 % CO_2_ incubator.

### Lentivirus and plasmid transfections

Lentivirus knockdown HMGA2, knockdown hnRNPK, knockdown YAP, overexpressing hnRNPK and overexpressing YAP were purchased from Genechem (Shanghai, China). Ubiquitin plasmid, HMGA2 full-length plasmids and three HMGA2 truncator plasmids were purchased from Genescript (Nanjing, China). All sequences were listed in Table S4. Lentivirus transfections were performed using HiTransG P (REVG005, Genechem) and puromycin (3 μg/mL) was used to select the positively-infected cells for 3 days. Plasmid transfections were performed using Lipofectamine 3000 (L3000008, Invitrogen).

### Transwell assays

4×10⁴ PC cells per chamber were added to the upper compartments, which were thereafter maintained in serum-free basal medium. Conversely, the lower chamber contained DMEM with 10% FBS. For the invasion assay, Matrigel (356234, Corning) was applied to the upper compartment prior to cell seeding. Following 24 hours of incubation, 4% paraformaldehyde and crystal violet were used to fix and stain migrated PC cells.

### Wound healing assay

PC cells were inoculated to 6-well plates and cultured until achieving ≥95% confluency. A pipette tip was used to generate the uniform wound in cell monolayer, with the initial wound area documented by microscopic photography. Following medium replacement with serum-free basal medium, images of wound area were captured at preset time points.

### Animal experiments

Six-week-old female C57BL/6 mice received 1×10^6^ KPC cells per mouse via splenic tissue injection for the implantation experiment. Two separate cohorts of mice were used following. Cohort 1 was humanely euthanized thirty days after injection, and body weight, liver weight and the count of metastatic nodules in the liver were recorded. Cohort 2 was dedicated to overall survival monitoring. Animals in the survival cohort were inspected daily, with survival data recorded and plotted as Kaplan-Meier curves. In the ciclopirox treatment experiment, mice were randomly assigned to two groups: NC (nontreated control) and Ciclopirox (gavage, 20 mg kg^-1^, every 2 days). Predefined humane endpoints were strictly followed: mice were sacrificed if body weight decreased by more than 20%, accompanied by persistent inactivity, ascites formation or difficulty moving.

### Co-Immunoprecipitation (Co-IP)

Protein A/G PLUS-Agarose (sc-2003, SANTA CRUZ) was used to conduct the Co-IP assay. Cells were lysed in ice-cold RIPA buffer, clarified, optionally pre-cleared with Protein A/G PLUS-Agarose and control IgG, incubated with primary antibody (1μg) and agarose beads (20μl) overnight at 4°C. Then agarose beads were washed and heated (95°C, 5 min). The eluates were ultimately analyzed by Western Blot.

### Proteomic analysis using LC-MS/MS

Coomassie-stained SDS-PAGE gels were used to excise target protein bands, which were then destained in a solution of 5% ACN and 25 mM NH_4_HCO_3_. Following 15-minute dehydration with 100% ACN, the samples were treated with 10 mM DTT, alkylated with 55 mM IAA, and digested overnight with trypsin. Then peptides extracted with 50% ACN/5% FA were concentrated by vacuum centrifugation, and subjected to LC-MS/MS analysis using a SCIEX ZenoTOF 7600 high-resolution tandem mass spectrometer. Initial searching of raw MS/MS data was completed using ProteinPilot Software v4.5 (SCIEX), and secondary analysis was implemented with Protein Prospector v5.19.1 (UCSF) against the Uniprot Human database (04/08/2019; 20,419/559,228 entries).

### CHX and proteasome inhibition assay

CHX and MG132 was purchased from MCE (Shanghai, China). PC cells were inoculated into 6-well plates at a uniform density and incubated for 24h. For the CHX assay, PC cells were treated with 50 μM CHX for 0h, 1h, 2h, 4h, 6h, 8h. For the proteasome inhibition assay, cells were treated with 20 μM MG132 for 4h. Protein half-life was assessed according to post-CHX treatment protein expression. Then proteins were extracted and subjected to Western blot to quantify expression levels.

### Chromatin immunoprecipitation (ChIP)

Pierce™ Magnetic ChIP Kit (26157, Thermo Fisher Scientific) was used to conduct the ChIP assay. Immunoprecipitation was carried out with target-specific antibodies (mouse IgG as negative control). Primers were constructed based on the sequences of YAP promoter, and the ChIP-qRT-PCR primer sequences are listed below: Yap chip-F: CCTTCAGAATTCTCCTGGGCA; Yap chip-R: CAGGCACCTGATGTACAAGGA. The resulting products were separated via 2% agarose gel electrophoresis to facilitate subsequent analytical procedures.

### Luciferase reporter assay

The plasmids carrying the firefly luciferase reporter gene and the YAP promoter sequence, as well as the plasmids carrying the renilla luciferase reporter gene were purchased from Genecreate (Wuhan, China). At 4h after transfection, PC cells were analyzed using Dual Luciferase Reporter Assay Kit (DL101-01, Vazyme) following the manufacturer's instructions.

### Statistical analysis

Statistical analysis was performed using GraphPad Prism (version 10.0.1). Differences among groups were assessed via Wilcoxon signed rank test or unpaired Student's t test (for 2 groups), one-way ANOVA followed by Tukey's test (for more than 2 groups), Chi-square test (for categorical clinicopathological variables) or Log-rank test (for survival). All data were presented as mean ± standard deviation (SD) for at least three independent samples. Statistical significance was established as *P < 0.05, **P < 0.01, and ***P < 0.001.

## Results

### LM of PC exhibits a distinct upregulated transcriptional signature

To identify genes associated with LM of PC, we collected primary PC tissues and matched LM tissues from PC patients diagnosed with LM at initial presentation and then performed RNA-seq (Fig. 1A). Since CTCs have been confirmed to be closely related to PC metastasis 18, we collected the peripheral blood of these PC patients and isolated CTCs (Fig. 1A). White blood cells (WBC) served as controls, IF staining revealed prominent expression of the surface markers CK and FRα of CTCs characterized in our previous study 19, confirming the successful enrichment of CTCs in the isolated cells (Fig. 1B). To expand the transcriptional profile across LM, we included a transcriptomic dataset consisting of matched CTCs and primary tumors from GSE118556. Subsequent analyses identified a shared set of differentially upregulated genes (n = 72) that are commonly enriched in LM and CTCs relative to their matched primary tumors in PC. Among these overlapping genes identified, HMGA2 exhibited a remarkably high ranking (Fig. 1C-E). Furthermore, enrichment analyses of KEGG pathways and GO terms demonstrated that HMGA2 was functionally integrated into key pathways and processes governing transcriptional regulation, cellular proliferation and cancer-related signaling, which indicated that HMGA2 might serve as a potential driver of LM in PC (Fig. 1F).

### HMGA2 correlates with LM and indicates unfavorable prognosis

To dissect whether HMGA2 elevation contributes to metastatic progression or arises from clonal selection, we interrogated public single-cell RNA-seq datasets containing paratumor, primary tumor and LM tissues (Fig. 2A and Fig. S1A). Further analysis revealed that HMGA2 expression was markedly increased in tumor epithelial cells of LM relative to primary tumor, while minimal HMGA2 signal was detected in paratumor (Fig. 2B, C). These data demonstrate that HMGA2 is transcriptionally upregulated within metastatic tumor cells, consistent with a potential role in liver dissemination. qPCR was then used to determine the mRNA expression of HMGA2 in PC, LM and CTCs samples. The results showed that HMGA2 was significantly upregulated in LM tissues (Fig. 2D). Furthermore, HMGA2 expression was barely detectable in WBC, while CTCs exhibited notably higher HMGA2 expression compared to PC tissues (Fig. 2E, F).

The results from database analysis also revealed that HMGA2 expression was increased in matched LM tissues relative to PC tissues, and HMGA2 was nearly undetectable in normal pancreatic tissues (Fig. 3A, B). We got the same results after collecting tissues from 5 PC patients for Western blot (Fig. 3C). A total of 10 post-surgical PC patients were then enrolled for regular follow-up and categorized into the LM group and non-liver metastasis (NonLM) group for 1 year, and their PC tissues were collected for Western blot detection, which revealed that HMGA2 expression was notably higher in the LM group compared with the NonLM group (Fig. 3D). Analysis of TCGA dataset also indicated that PC patients with elevated HMGA2 expression presented remarkably shorter overall survival (OS) and disease-free survival (DFS) (Fig. 3E, F). Furthermore, based on tissue microarray cohort consisting of 61 PC patients, we investigated the clinical relevance of HMGA2 expression (Fig. 3G). Kaplan-Meier survival analysis showed that elevated HMGA2 expression was significantly correlated with poorer OS in PC patients (Fig. 3H). Further clinicopathological correlation analysis indicated that elevated HMGA2 expression was significantly associated with distant metastasis (M1 stage) and advanced TNM stage (Table S5).

Subsequently, we analyzed another public single-cell RNA-seq dataset containing 24 PC tissues and 11 normal pancreatic tissues, and the findings revealed that HMGA2 was significantly more highly expressed in PC tissues than in normal counterparts (Fig. S1B, C), with its expression closely correlating with PC stage and cancer subtype (Fig. S1D, E). Further analysis after dividing the cells into 10 types based on the gene expression of cell type markers showed that tumor cells exhibited significantly higher HMGA2 expression compared to pancreatic acinar cells and normal pancreatic ductal cells (Fig. S1F, G). Taken together, these findings indicate that high HMGA2 expression may drive PC cells to enter the bloodstream as CTCs and progress to LM of PC.

### HMGA2 knockdown restrains liver metastatic capacity of PC *in vitro* and *in vivo*

To investigate HMGA2's function in PC progression, lentivirus-HMGA2-shRNA was used to silence HMGA2 in PANC-1 and KPC cells both of which exhibit high HMGA2 expression, with Western blot confirming efficient knockdown (Fig. 4A and Fig. S2A, B). Since EMT is a well-recognized key driver of tumor cell invasion and metastasis 20, we analyzed EMT-related markers, and the results of Western blot revealed that HMGA2 knockdown notably reduced mesenchymal markers (Vimentin, N-cadherin) while upregulating epithelial marker E-cadherin in PC cells (Fig. 4B and Fig. S2C).

Transwell migration and invasion assays were then conducted, revealing that HMGA2 knockdown significantly attenuated the migratory and invasive potential of PC cells (Fig. 4C, D and Fig. S2D, E). Similarly, wound healing assay also demonstrated HMGA2 knockdown significantly expanded the wound width of PC cells (Fig. 4E, F and Fig. S2F, G). Collectively, these findings support HMGA2's pro-metastatic function in tumor cells under *in vitro* conditions.

To explore the regulatory role of HMGA2 on LM *in vivo*, we performed intrasplenic injection of KPC cells with stable HMGA2 knockdown into C57BL/6 mice (Fig. 4G, H). After thirty days of KPC cells injection, our results demonstrated that HMGA2 knockdown significantly reduced liver weight and the count of liver metastatic nodules in mice, with no notable differences in body weight relative to the shNC group (Fig. 4I-M). Meanwhile Kaplan-Meier analysis revealed that one group of shHMGA2 mice had a significantly shortened survival time of than that in the shNC group (Fig. 4N). Collectively, these results confirm that HMGA2 actively contributes to the promotion of LM *in vivo*, consistent with our *in vitro* observations.

### HMGA2 binds to and co-localizes with hnRNPK

Given that one of HMGA2's key functions is to interact with other proteins, we enriched HMGA2 interacting proteins in PC cells through Co-IP assay, and then subjected them for protein identification through proteomic analysis (Fig. 5A). LC-MS/MS analysis results demonstrated that the peptide count of hnRNPK ranked high among the identified proteins which could bind with HMGA2, and multiple peptide sequences of hnRNPK were identified (Fig. 5B-D). Although mass spectrometry screening of HMGA2 immunoprecipitation complexes detected multiple hnRNP family proteins, all other hnRNP paralogs including hnRNPH2, hnRNPR and hnRNPD only showed low background enrichment with far fewer unique peptides, demonstrating preferential protein-protein binding between HMGA2 and hnRNPK (Table S6). Western blot of Co-IP assay further confirmed that HMGA2 interacted with hnRNPK in PC cells (Fig. 5E, F). We then fluorescent labeled the HMGA2 protein and hnRNPK protein by IF staining, and found that HMGA2 and hnRNPK were co-located in the nucleus of PC cells (Fig. 5G). Taken together, these results indicate that HMGA2 co-locates and interacts with hnRNPK.

To further investigate which domain of HMGA2 directly interacts with hnRNPK, we then designed HMGA2 full-length (FL) plasmids with HA tags as well as three different HMGA2 truncator plasmids, HMGA2 (1-83), HMGA2 (1-73) and HMGA2 (36-109) based on the amino acid sequence of HMGA2 protein and transfected them into HEK293T cells (Fig. 5H). Subsequently, HA-tagged proteins were enriched via Co-IP assay and hnRNPK expression was detected by Western blot analysis. The results demonstrated that HMGA2 FL, HMGA2 (1-83) and HMGA2 (36-109) were all capable of binding to hnRNPK, whereas HMGA2 (1-73) failed to form such an interaction (Fig. 5I). These results imply that the 73-83 amino acid sequence domain of HMGA2 could be pivotal for its binding activity toward hnRNPK, which corresponds to the protein's “AT-hook” functional motif.

### HMGA2 suppresses hnRNPK ubiquitination to block proteasomal degradation

To clarify the regulatory interplay between HMGA2 and hnRNPK, we evaluated the expression levels of hnRNPK following HMGA2 knockdown in PC cells. Notably, the results revealed a concurrent downregulation of hnRNPK expression, which suggests that there may be a positive regulatory effect of HMGA2 on hnRNPK (Fig. 6A, B). TCGA database-derived results demonstrated that the Spearman correlation coefficient (R) between HMGA2 and hnRNPK mRNA expression levels in PC tissues was 0.24, corresponding to an almost non-existent correlation (Fig. 6C), which means that HMGA2 may not regulate hnRNPK through transcription or at the RNA level. Considering that we have demonstrated that HMGA2 is able to bind and interact with hnRNPK, we speculate that HMGA2 might regulate hnRNPK expression at the protein level.

To verify the above conjecture, we treated PC cells in shNC group and shHMGA2 group with 50 μM CHX for 0h, 1h, 2h, 4h, 6h and 8h respectively. The results demonstrated that hnRNPK protein expression in shHMGA2 group exhibited a time-dependent decrease, with levels dropping to 50% of the baseline at around 4 hours. In contrast, hnRNPK protein levels remained largely unchanged in shNC group throughout the experimental period (Fig. 6D, E). To further clarify if the proteasome system mediates HMGA2-dependent regulation of hnRNPK protein levels, we treated shNC and shHMGA2 cells with proteasome inhibitor MG132 or DMSO for 4 hours respectively. The results demonstrated that DMSO-treated shHMGA2 cells exhibited a significant decrease in hnRNPK protein expression compared with the shNC group. However, MG132 treatment alleviated the downregulation of hnRNPK protein in shHMGA2 cells, resulting in a lesser reduction in expression levels (Fig 6F, G). We next conducted ubiquitination assays to verify whether HMGA2 regulates hnRNPK ubiquitination. The Western blot results demonstrated that ectopic HMGA2 expression significantly reduced the ubiquitinated fraction of hnRNPK in HEK293T cells, while HMGA2 knockdown markedly increased the ubiquitination level of endogenous hnRNPK in PANC-1 cells (Fig. 6H, I). Taken together, these findings indicate that HMGA2 interacts with hnRNPK to improve hnRNPK protein stability, ultimately inhibiting its ubiquitin-proteasome-dependent degradation.

### HMGA2 promotes LM of PC via modulating the expression of hnRNPK

To verify whether HMGA2 promotes LM of PC by regulating hnRNPK, we knocked down hnRNPK in PANC-1 and KPC cells through lentivirus-hnRNPK-shRNA, and overexpressed hnRNPK in shHMGA2 cells. Western blot analysis confirmed the knockdown and overexpression efficiency (Fig. 7A and Fig. S3A). Results from Transwell and wound healing assays demonstrated that knockdown hnRNPK markedly reduced the migration and invasion abilities of PC cells, while hnRNPK overexpression reversed the HMGA2 knockdown-induced inhibition of these metastatic phenotypes (Fig. 7B-E and Fig. S3B-E).

Similarly in animal experiments, we injected the above 4 types of PC cells into C57BL/6 mice through spleen to build LM model (Fig. S3F). The results demonstrated that neither hnRNPK knockdown nor overexpression had an impact on mouse body weight. In contrast, hnRNPK knockdown group exhibited a significant reduction in liver weight and the count of liver metastatic nodules, which indicated that the knockdown of hnRNPK inhibits the formation of LM in mice. Besides, overexpression of hnRNPK reversed the inhibition of LM in mice induced by HMGA2 knockdown (Fig. 7F-J). Collectively, our *in vitro* and *in vivo* data validated the functional relevance of the HMGA2-hnRNPK regulatory axis in LM of PC.

### hnRNPK binds to YAP promoter to activate its transcription

hnRNPK has been confirmed to function as a transcription factor 21. Using a candidate-gene approach, we curated a panel of well-established metastasis-related transcriptional regulators, including YAP, STAT3, MYC, SNAI1, TWIST1, CTNNB1, ZEB1, FOXM1 and SOX9, and sequentially calculated their pairwise Spearman correlations with hnRNPK in the PC cohort to prioritize potential downstream targets. The results revealed that YAP exhibited the strongest positive co-expression correlation with hnRNPK (Fig. 8A and Fig. S4A). Consistent results were further validated by the Similar Genes function of GEPIA2, where YAP ranked among the top positively correlated genes of hnRNPK in PC (Fig. S4B). Western blot demonstrated that hnRNPK downregulation in PC cells led to a concurrent downregulation of YAP expression (Fig. 8B). Therefore, we hypothesized that hnRNPK might activate YAP through transcription and ultimately affect its protein expression in PC cells. We then designed lentiviruses knocking down and overexpressing YAP to be transfected in PC cells and identified the knockdown and overexpression efficiencies by Western blot for subsequent validation experiments (Fig. 8B). In addition, we found the expression of hnRNPK and YAP was synchronously downregulated in shHMGA2 cells, but overexpression of hnRNPK after HMGA2 knockdown led to the re-upregulation of YAP expression (Fig. 8C, D), which indicated that YAP might be a potential target for regulation after HMGA2 binding and interacting with hnRNPK.

To verify whether hnRNPK regulates YAP expression at the transcriptional level, we performed ChIP experiments to enrich DNA bound by hnRNPK protein and then verified by agarose gel electrophoresis and qPCR respectively. The results demonstrated that YAP promoter activity was markedly elevated in the Anti-hnRNPK group relative to the Anti-IgG control group, suggesting that hnRNPK interacts with the YAP gene's promoter region to drive its transcriptional activation. Conversely, HMGA2 knockdown inhibited the binding of hnRNPK to the YAP promoter by downregulating hnRNPK protein level (Fig. 8E, F). In addition, luciferase reporter assay results indicated that HMGA2 or hnRNPK knockdown suppressed YAP promoter activity, while hnRNPK overexpression abrogated the inhibitory effect of HMGA2 knockdown on YAP promoter-driven transcription (Fig. 8G). Furthermore, we found that overexpression of YAP reversed the inhibition of hnRNPK knockdown on EMT of PANC-1 cells (Fig. 8H).

### YAP serves as the downstream effector of the HMGA2-hnRNPK axis

To validate that YAP acts as the essential downstream effector mediating HMGA2-hnRNPK-driven LM, we conducted two parallel sets of knockdown-rescue functional assays *in vitro* and *in vivo*. Western blots confirmed stable HMGA2 or hnRNPK knockdown and successful YAP overexpression in respective rescue groups (Fig. S5A and Fig.S6A). Wound healing and Transwell assays uniformly showed knockdown of either HMGA2 or hnRNPK suppressed cell migration and invasion, which was fully rescued by YAP overexpression *in vitro* (Fig. S5B-E and Fig.S6B-E). *In vivo* results recapitulated this rescue phenotype: shHMGA2 and shhnRNPK groups both exhibited reduced liver weight and fewer metastatic nodules without altered body weight, while YAP overexpression completely restored hepatic metastatic capacity (Fig. S5F-I and Fig.S6F-I). Taken together, these dual rescue experiments establish YAP as the downstream mediator transmitting pro-metastatic signals from the HMGA2-hnRNPK cascade.

Finally, to assess the therapeutic value of targeting HMGA2 in PC, we intrasplenically transplanted KPC cells into mice and treated them with ciclopirox (a HMGA2 inhibitor) 22 (Fig. 8I). The results showed that the treatment of ciclopirox suppressed LM, and no obvious body weight loss was observed during the gavage treatment period (Figure 8J-M). We further isolated liver metastatic tissues from NC and ciclopirox-treated mice for Western blot detection. The results revealed that ciclopirox treatment was accompanied by reduced expression of HMGA2, hnRNPK and YAP, consistent with suppression of the HMGA2/hnRNPK-YAP axis *in vivo* (Fig. 8N). In conclusion, our results suggest that HMGA2 interacts with hnRNPK to form a functional complex, which enhances hnRNPK's binding affinity to the YAP promoter region and ultimately promotes LM of PC (Fig. 8O).

## Discussion

In-depth exploration of the biological characteristics and pathogenesis of LM of PC, coupled with the discovery of appropriate molecular targets, is imperative to achieve early clinical diagnosis and develop effective treatment strategies for this lethal malignancy. In this study, we found that HMGA2 exhibits increased expression in LM tissues and CTCs, and HMGA2 knockdown inhibits the LM progression of PC. Elevated expression of HMGA2 may be a driver of LM, which cooperates with hnRNPK to activate YAP, promoting PC cells to enter into the bloodstream from the primary site to become CTCs and eventually colonize the liver. Notably, our *in vitro* functional assays demonstrate that loss of HMGA2 markedly attenuates PC cell migration and invasion potential, which are prerequisites for primary tumor cells to enter the portal circulation. However, these assays do not directly recapitulate intravasation, and whether HMGA2 specifically promotes this step remains to be determined. Furthermore, our intrasplenic injection model bypasses primary tumor invasion and primarily evaluates hepatic colonization and outgrowth of disseminated cells; the reduced metastatic burden upon HMGA2 knockdown in this model therefore supports a role for HMGA2 in liver colonization and survival of disseminated cells, rather than intravasation per se. The elevated HMGA2 expression observed in patient CTCs is consistent with a potential function in CTCs survival, but this remains associative and requires functional validation. Taken together, our data establish that HMGA2 promotes PC cell invasion *in vitro* and hepatic colonization *in vivo*, and we propose that the HMGA2/hnRNPK-YAP axis may additionally support CTCs survival during hematogenous dissemination. Dissecting the precise contribution of HMGA2 to each sequential step of the metastatic cascade, particularly intravasation and CTCs survival, will require orthotopic implantation models and dedicated *in vivo* assays in future studies.

As a regulatory protein belonging to the hnRNP family, hnRNPK is pivotal for cancer metastasis via driving cell motility, enhancing tumor angiogenesis and modulating extracellular matrix remodeling 23. Fallatah et al. found that K19 directly interacts with hnRNPK and prompts hnRNPK to shuttle to the cytoplasm further promoting cell proliferation and breast cancer metastasis 24. Another study showed that PROX1 interacts with hnRNPK to inhibit its ubiquitination, and this stabilization of hnRNPK activates the WNT signaling pathway, ultimately driving breast cancer invasion and metastasis 25. In addition, HDM2 binding to hnRNPK induces its ubiquitination and promotes its degradation through the proteasome pathway 26, 27. Furthermore, it has been reported that FBXW7 interacting with hnRNPK phosphorylates hnRNPK at the threonine 1695 site to promote degradation, and enhances the malignant biological behaviors of PC cells 28. In this study, through LC-MS/MS, IF staining, CHX assay and proteasome inhibition assay we found that HMGA2 binds to hnRNPK and co-localizes in the nucleus of PC cells, and the main role is played by the third segment of HMGA2's “AT-hook” functional motif. The ubiquitination assay confirms that HMGA2 suppresses hnRNPK ubiquitination to prevent its proteasomal turnover. Other hnRNP family members lack the structural motif that enables HMGA2 interaction, resulting in their minimal enrichment in our HMGA2 Co-IP mass spectrometry data. The interaction between HMGA2 and hnRNPK enhances the stability of hnRNPK protein and finally regulates LM of PC.

Beyond its protein-binding capacity, hnRNPK acts as a transcription factor which mediates the transcriptional activity of target genes 29. Notably, the Hippo signaling pathway is well-established as a pivotal mediator of cancer metastatic progression, with its functional relevance to PC metastasis being tightly linked to the modulation of YAP activity 30, 31. A recent study in non-small cell lung cancer demonstrated that hnRNPK upregulates YAP's mRNA and protein expression as well as its transcriptional activity, with siRNA-mediated YAP knockdown abrogating hnRNPK's stimulatory effect on H1299 cell proliferation 32. Another study showed that LINC01413 binds to hnRNPK and induces YAP nuclear translocation, ultimately enhancing colorectal cancer cell migration, invasion and EMT 33. Furthermore, lnc - CTHCC directly interacts with hnRNPK and facilitates the development and progression of hepatocellular carcinoma by activating YAP transcription 34. These results suggest that YAP is an effector of hnRNPK. Under pathological conditions, impaired Hippo kinase signaling reduces YAP phosphorylation, facilitating YAP nuclear translocation to drive cell proliferation, suppress apoptosis, maintain stem cell properties and promote migration and invasion 35. Our study identifies an independent transcriptional regulatory module upstream of YAP that does not interfere with YAP phosphorylation status. Mechanistically, HMGA2 interacts with hnRNPK and suppresses hnRNPK ubiquitination, preventing its proteasomal degradation and enriching cellular hnRNPK pools. Accumulated hnRNPK directly binds the YAP gene promoter to boost YAP transcription, elevating total YAP protein levels. However, this HMGA2-hnRNPK transcriptional pathway may also cooperate with the Hippo cascade, collectively amplifying YAP-dependent LM programs in PC. Furthermore, as an effector molecule downstream of hnRNPK, YAP serves a critical function in mediating the regulatory modulation of EMT.

Several limitations should be acknowledged in this study. First, although we have validated that ciclopirox exerts anti-LM effects by targeting HMGA2, this agent is originally developed as a broad-spectrum antifungal drug, and its anti-tumor effects are multi-faceted beyond HMGA2 suppression: the compound acts as an iron chelator to disrupt iron-dependent proliferative signaling, additionally inhibiting Wnt, EGFR/Akt and other oncogenic cascades in digestive malignancies, which may create potential off-target impacts *in vivo* 36. Second, we only validated the anti-metastatic activity of ciclopirox in intrasplenic xenograft models, but its specificity and efficacy in orthotopic models should be explored further. Moreover, we did not conduct parallel animal experiments with other HMGA2-targeted agents such as netropsin and distamycin A, nor did we perform *in vivo* CRISPR-Cas9-mediated HMGA2 knockout for orthogonal verification, and we will incorporate animal studies on drug and genetic interventions for further exploration. Last, we have not clarified whether HMGA2 attenuates hnRNPK ubiquitination by competitively blocking the recruitment of E3 ubiquitin ligases or by facilitating the assembly of deubiquitinase-hnRNPK complexes, and mass spectrometry profiling of HMGA2-interacting ubiquitin-related enzymes will be needed to resolve this detailed molecular event.

In conclusion, our findings suggest that HMGA2 co-localizes with hnRNPK in the nucleus and can bind and interact with each other, repressing its ubiquitin-mediated degradation. hnRNPK subsequently activates the YAP promoter to induce YAP transcription, ultimately facilitating LM of PC. Our study seeks to illuminate the critical function of HMGA2 in regulating LM and provide a robust foundation for HMGA2/hnRNPK-YAP axis-targeted therapies, thereby opening new avenues for improving clinical management of PC patients with LM.

## Supplementary Material

Supplementary figures and tables.

## Figures and Tables

**Figure 1:**
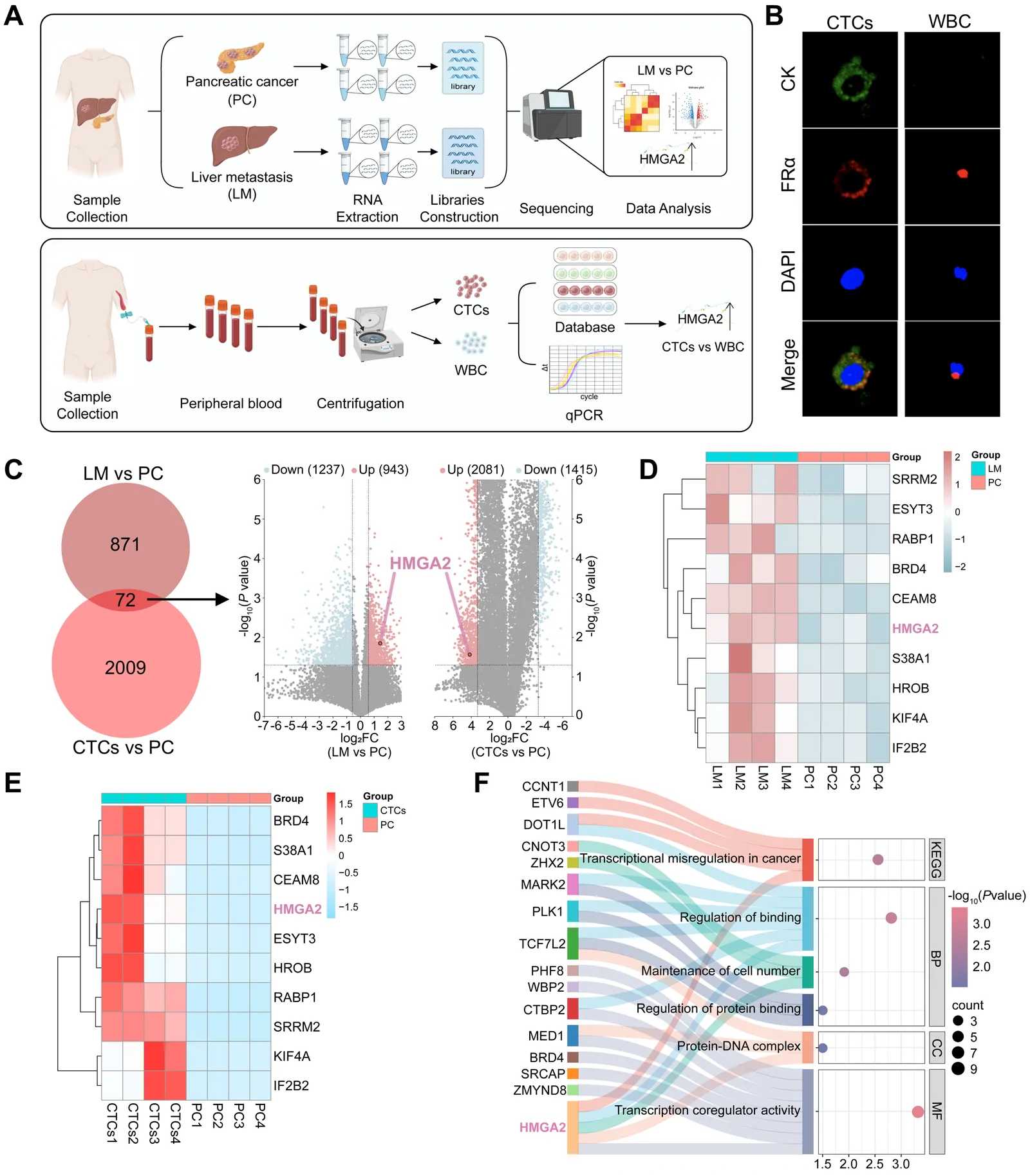
** LM of PC exhibits a distinct upregulated transcriptional signature. (A)** Schematic workflow of RNA-seq and CTCs experiments in this study. All tissues analyzed were obtained from 4 independent PC patients complicated with LM. **(B)** IF staining of CK and FRα in CTCs and WBC. **(C)** Venn diagram and Volcano plots demonstrating common and unique differentially upregulated genes between LM or CTCs and PC group. **(D)** Heatmap displaying top 10 differentially expressed genes between LM and PC group. **(E)** Heatmap displaying top 10 differentially expressed genes between CTCs and PC group. **(F)** KEGG pathway and GO enrichment analysis of jointly upregulate genes.

**Figure 2:**
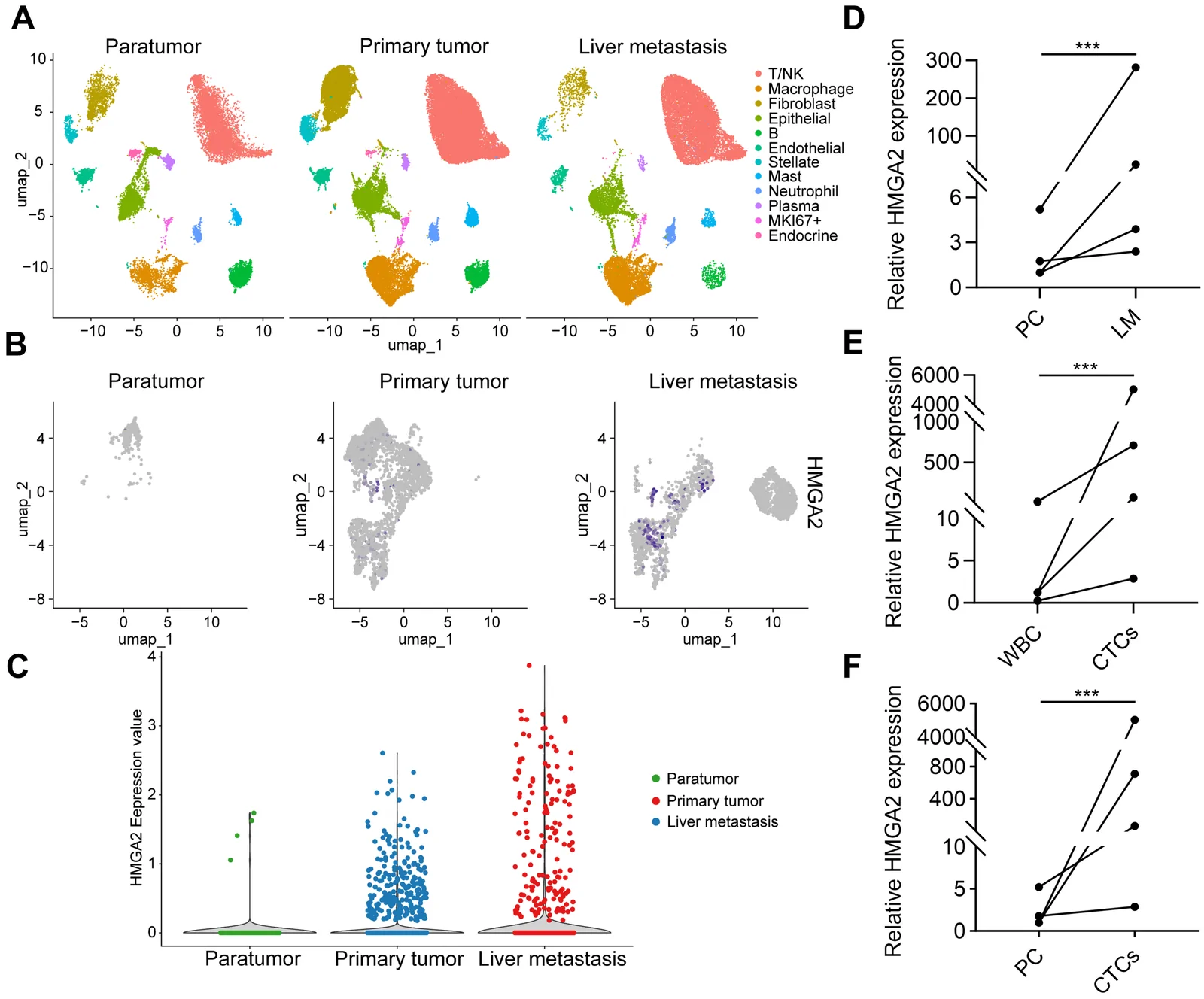
** The expression analysis of HMGA2 across different tissues of PC patients. (A)** UMAP plots show cell type annotation in epithelial tumor cells from three tissue groups. **(B)** UMAP plots show HMGA2 feature expression in epithelial tumor cells from three tissue groups. **(C)** Violin plot quantitatively compares HMGA2 expression levels of tumor epithelial cells among three tissue groups. **(D)** qPCR analysis of HMGA2 mRNAs in LM and PC tissues (n = 3 independent experiments). **(E)** qPCR analysis of HMGA2 mRNAs in CTCs and WBC (n = 3 independent experiments). **(F)** qPCR analysis of HMGA2 mRNAs in CTCs and PC tissues (n = 3 independent experiments). Data are shown as mean ± SD. ***P < 0.001. Statistical significance was calculated using Wilcoxon signed rank test (for 2 groups) or one-way ANOVA followed by Tukey's test (for 3 groups).

**Figure 3:**
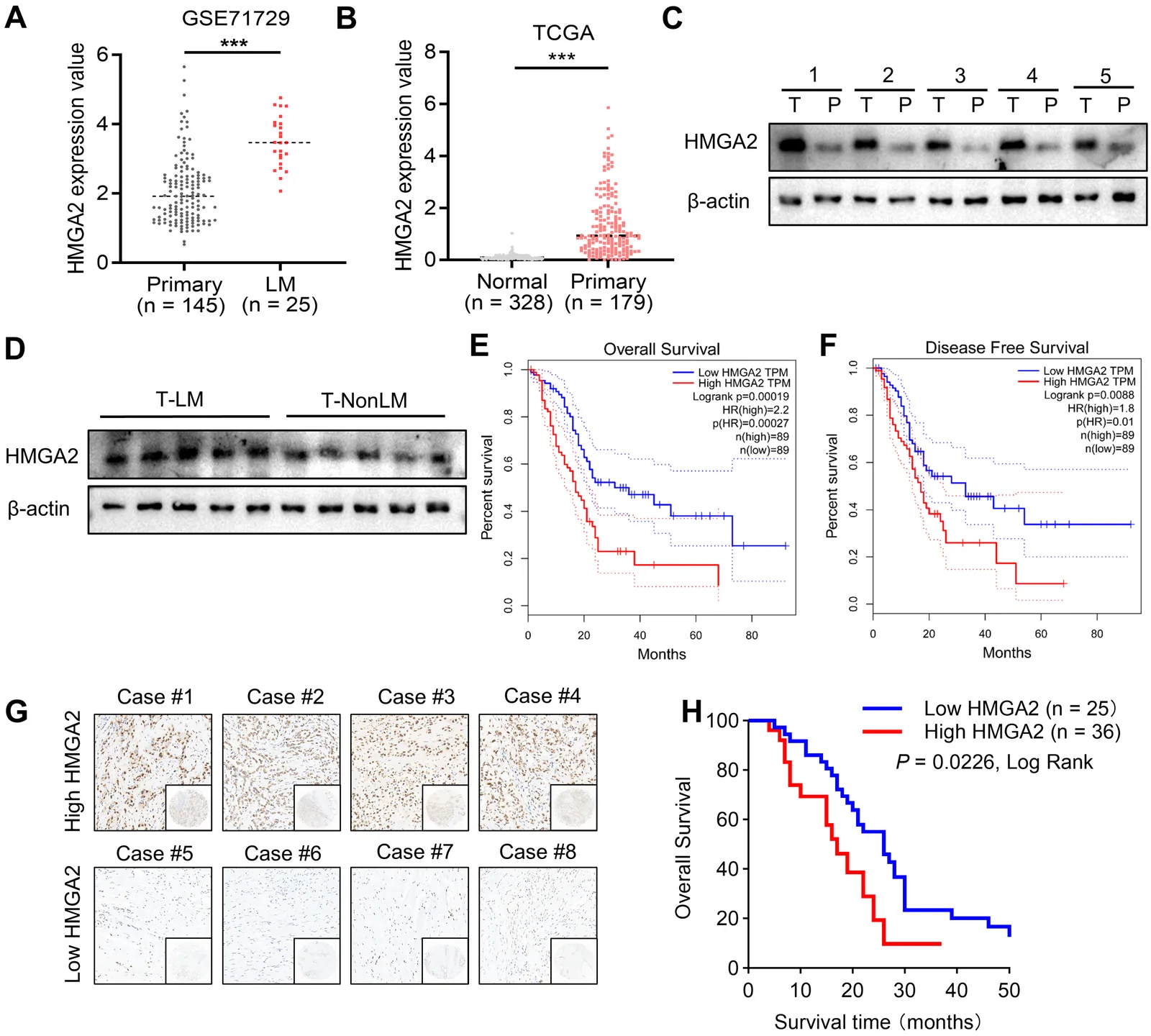
** HMGA2 correlates with LM and indicates unfavorable prognosis. (A)** Scatter plots of HMGA2 expression levels in primary tumor tissues and matched LM tissues. **(B)** Scatter plots of HMGA2 expression levels in primary tumor tissues and matched normal pancreatic tissues. **(C)** Western blot of HMGA2 expression in primary tumor tissues and matched paratumor tissues. **(D)** Western blot of HMGA2 expression in primary tumor tissues of LM and NonLM group. **(E)** OS analysis of PC patients in TCGA cohort stratified by HMGA2 expression levels. **(F)** DFS analysis of PC patients in TCGA cohort stratified by HMGA2 expression levels. **(G)** Representative IHC staining images of HMGA2 on the human PC tissue microarray. **(H)** OS analysis of PC patients in the human PC tissue microarray stratified by HMGA2 expression levels. Data are shown as mean ± SD. ***P < 0.001. Statistical significance was calculated using unpaired Student's t test (for 2 groups) or Log-rank test (for survival).

**Figure 4:**
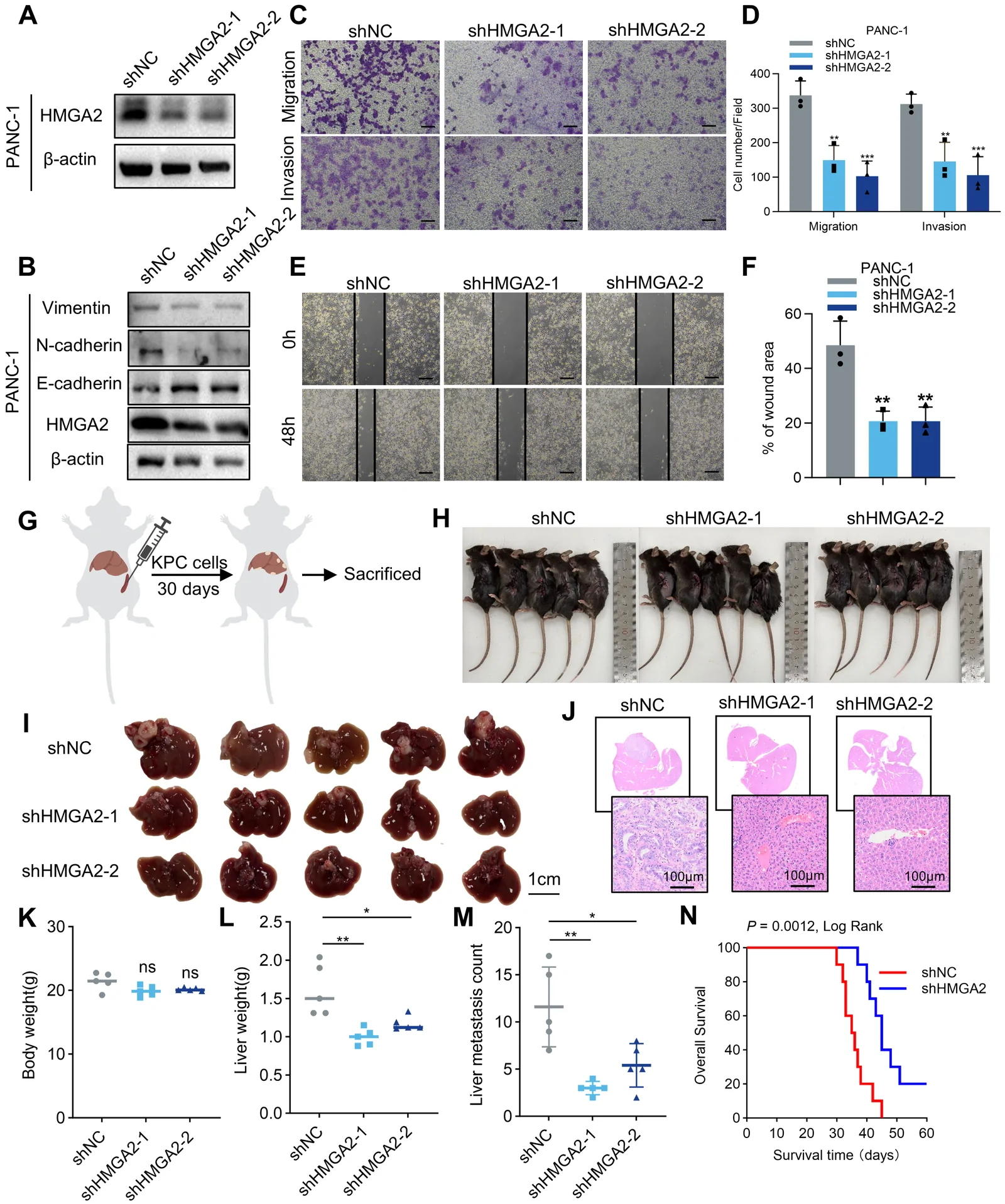
** HMGA2 knockdown restrains liver metastatic capacity of PC *in vitro* and *in vivo* (A)** Western blot of HMGA2 expression in shNC, shHMGA2-1 and shHMGA2-2 PANC-1 cells. **(B)** Western blot of EMT markers expression in shNC, shHMGA2-1 and shHMGA2-2 PANC-1 cells. **(C, D)** Representative images of Transwell assays and quantification of the number for shNC, shHMGA2-1 and shHMGA2-2 PANC-1 cells (n = 3 independent experiments). **(E, F)** Representative images of wound healing assay and quantification of the percentage of wound area for shNC, shHMGA2-1 and shHMGA2-2 PANC-1 cells (n = 3 independent experiments). **(G)** Experimental scheme of the *in vivo* LM model: Six-week-old female C57BL/6 mice received intrasplenic injection of KPC cells. **(H)** Mouse images of shNC, shHMGA2-1 and shHMGA2-2 groups. **(I)** Liver images of shNC, shHMGA2-1 and shHMGA2-2 groups. **(J)** Representative HE images of liver of shNC, shHMGA2-1 and shHMGA2-2 groups. **(K-M)** Mouse body weight (K), liver weight (L) and liver metastatic nodules count (M) analysis of shNC, shHMGA2-1 and shHMGA2-2 groups (n = 5 mice per group). (N) Kaplan-Meier overall survival curves generated from an independent separate cohort of mice subjected to identical intrasplenic injection procedure and long-term survival observation (n = 10 mice per group). Data are shown as mean ± SD. *P < 0.05, **P < 0.01, ***P < 0.001. Statistical significance was calculated using one-way ANOVA followed by Tukey's test (for 3 groups) or Log-rank test (for survival).

**Figure 5:**
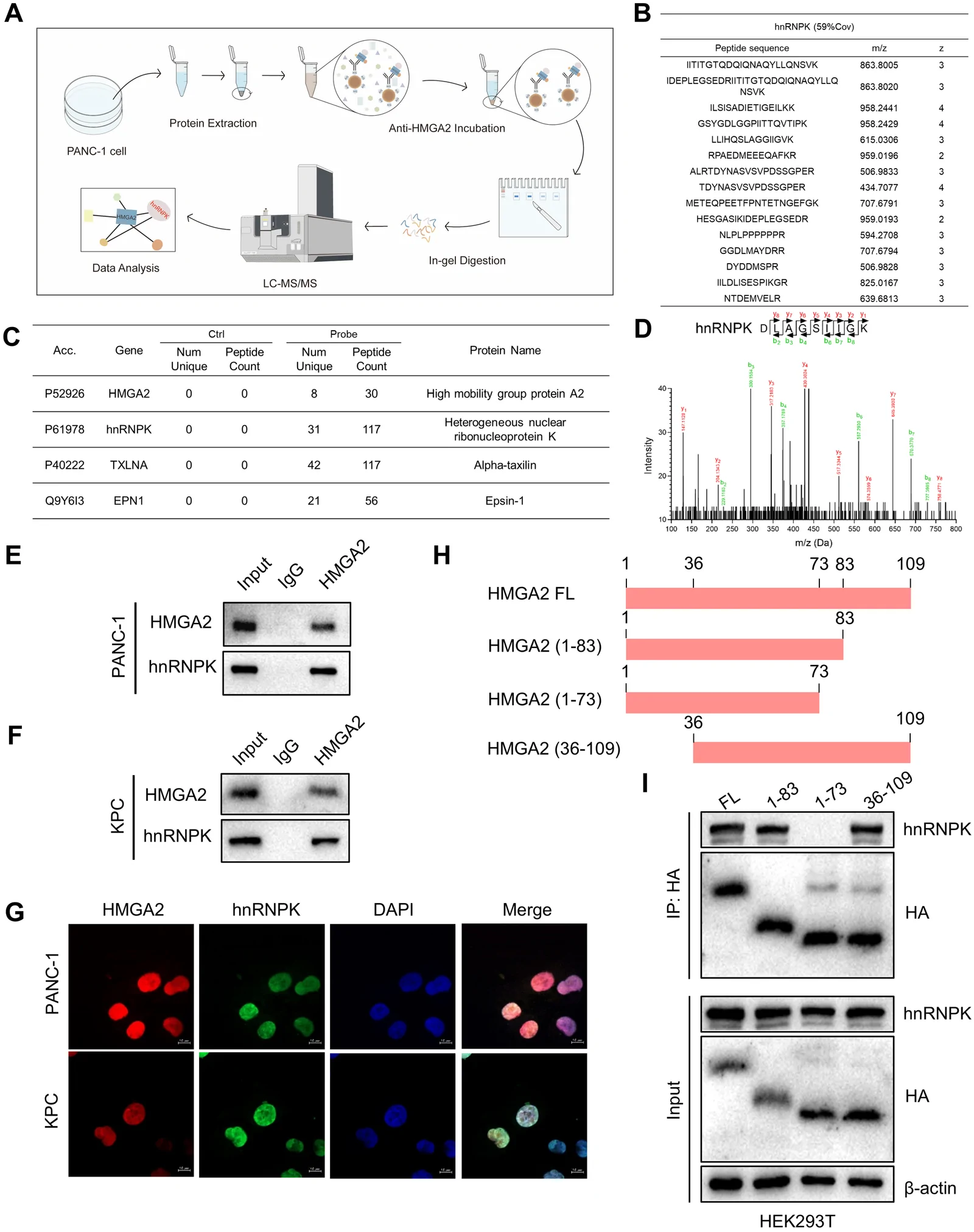
** HMGA2 binds to and co-localizes with hnRNPK. (A)** Schematic workflow of LC-MS/MS analysis in this study. **(B)** Unique peptide sequences detected of hnRNPK from the gel band of the immuno-precipitated fraction were identified using Proteinprospecter software. **(C)** hnRNPK was screened as an interesting protein among binding proteins for HMGA2 identified by LC-MS/MS. **(D)** Representative MS/MS spectra of unique peptides identified for hnRNPK. **(E)** Western blot of HMGA2 and hnRNPK expression following Co-IP assay in PANC-1 cells. **(F)** Western blot of HMGA2 and hnRNPK expression following Co-IP assay in KPC cells. **(G)** Representative images of IF staining of HMGA2 and hnRNPK in PC cells. **(H)** Diagram showing the truncation mutants (1-83, 1-73 and 36-109) and FL of HMGA2. The expected molecular weights of the HA-tagged constructs are approximately 12.9 kDa (FL), 10.0 kDa (1-83), 8.8 kDa (1-73), and 9.0 kDa (36-109). **(I)** Co-IP of the HMGA2 protein by an anti-HA antibody in HEK293T cells transfected with FL or various truncation mutants.

**Figure 6:**
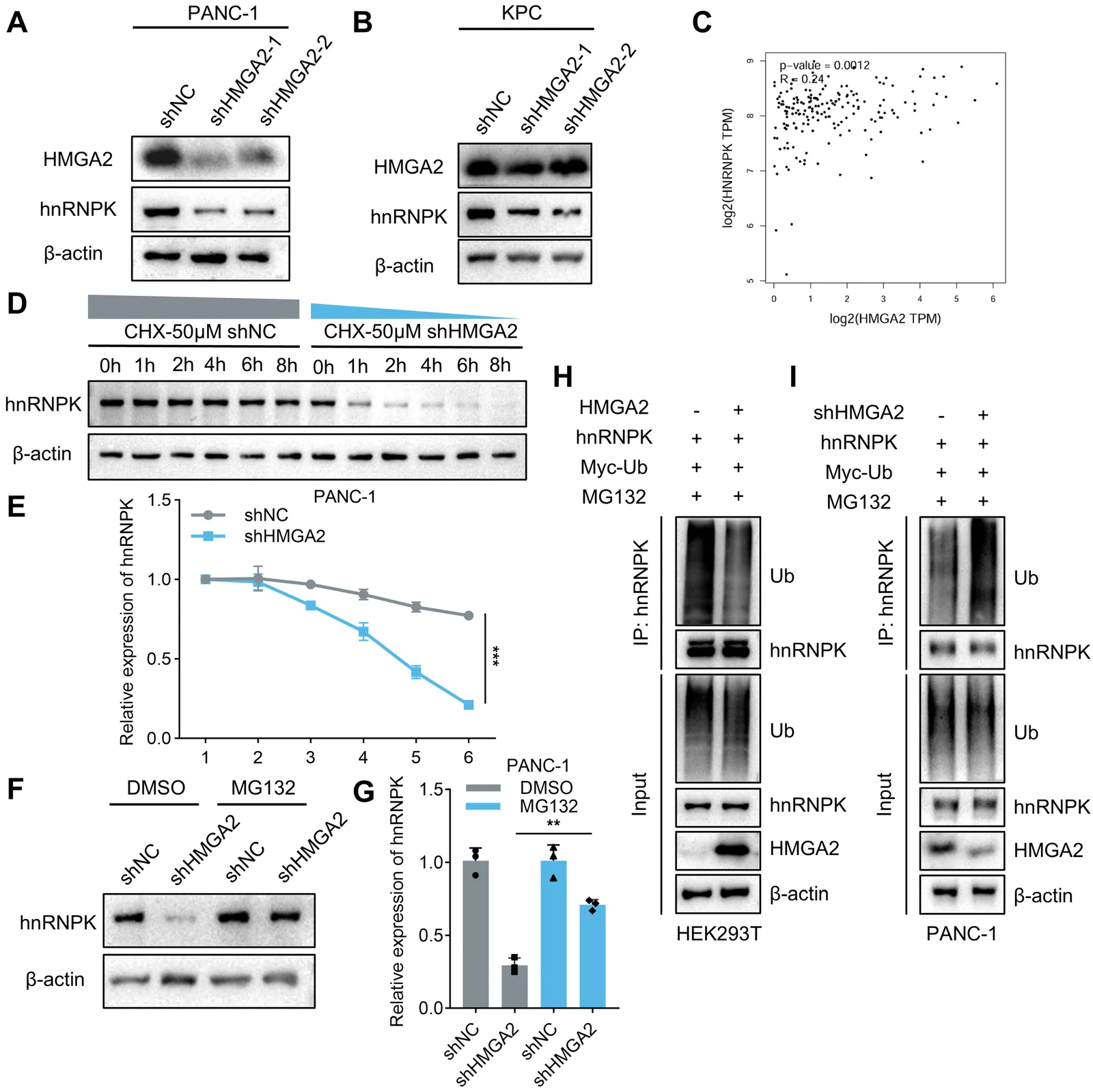
** HMGA2 suppresses hnRNPK ubiquitination to block proteasomal degradation. (A)** Western blot of HMGA2 and hnRNPK expression in shNC, shHMGA2-1 and shHMGA2-2 PANC-1 cells. **(B)** Western blot of HMGA2 and hnRNPK expression in shNC, shHMGA2-1 and shHMGA2-2 KPC cells. **(C)** Spearman correlation analysis between HMGA2 and hnRNPK in PC tissues. **(D, E)** Western blot and quantification of hnRNPK expression in shNC and shHMGA2 PANC-1 cells treated with CHX for 0 h, 1 h, 2 h, 4h, 6 h, 8 h (n = 3 independent experiments). **(F, G)** Western blot and quantification of hnRNPK expression in shNC and shHMGA2 PANC-1 cells treated with MG132 (n = 3 independent experiments). **(H)** Western blot of Ub, hnRNPK and HMGA2 expression in Input and IP groups of HEK293T cells after MG132 treated. **(I)** Western blot of Ub, hnRNPK and HMGA2 expression in Input and IP groups of PANC-1 cells after MG132 treated. Data are shown as mean ± SD. **P < 0.01, ***P < 0.001. Statistical significance was calculated using unpaired Student's t test (for 2 groups).

**Figure 7:**
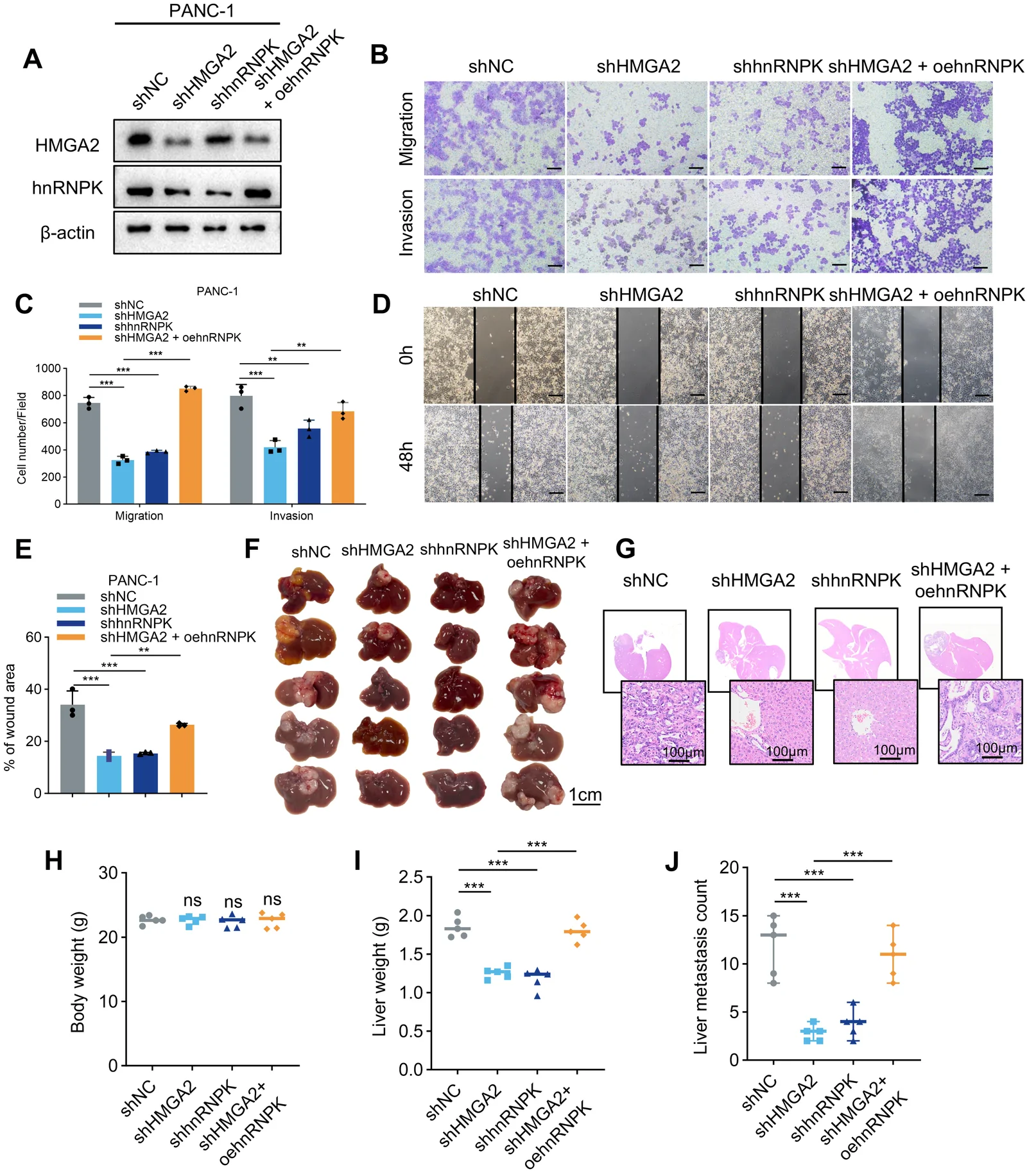
** hnRNPK overexpression reverses the inhibition of LM induced by HMGA2 knockdown. (A)** Western blot of HMGA2 and hnRNPK expression in shNC, shHMGA2, shhnRNPK and shHMGA2 + oehnRNPK PANC-1 cells. **(B, C)** Representative images of Transwell assays and quantification of the number for PANC-1 cells (n = 3 independent experiments). **(D, E)** Representative images of wound healing assay and quantification of the percentage of wound area for PANC-1 cells (n = 3 independent experiments). **(F)** Liver images of shNC, shHMGA2, shhnRNPK and shHMGA2 + oehnRNPK groups. **(G)** Representative HE images of liver of shNC, shHMGA2, shhnRNPK and shHMGA2 + oehnRNPK groups. **(H-J)** Mouse body weight (H), liver weight (I) and liver metastatic nodules count (J) analysis of shNC, shHMGA2, shhnRNPK and shHMGA2 + oehnRNPK groups (n = 5 mice per group). Data are shown as mean ± SD. **P < 0.01, ***P < 0.001. Statistical significance was calculated using one-way ANOVA followed by Tukey's test (for 4 groups).

**Figure 8:**
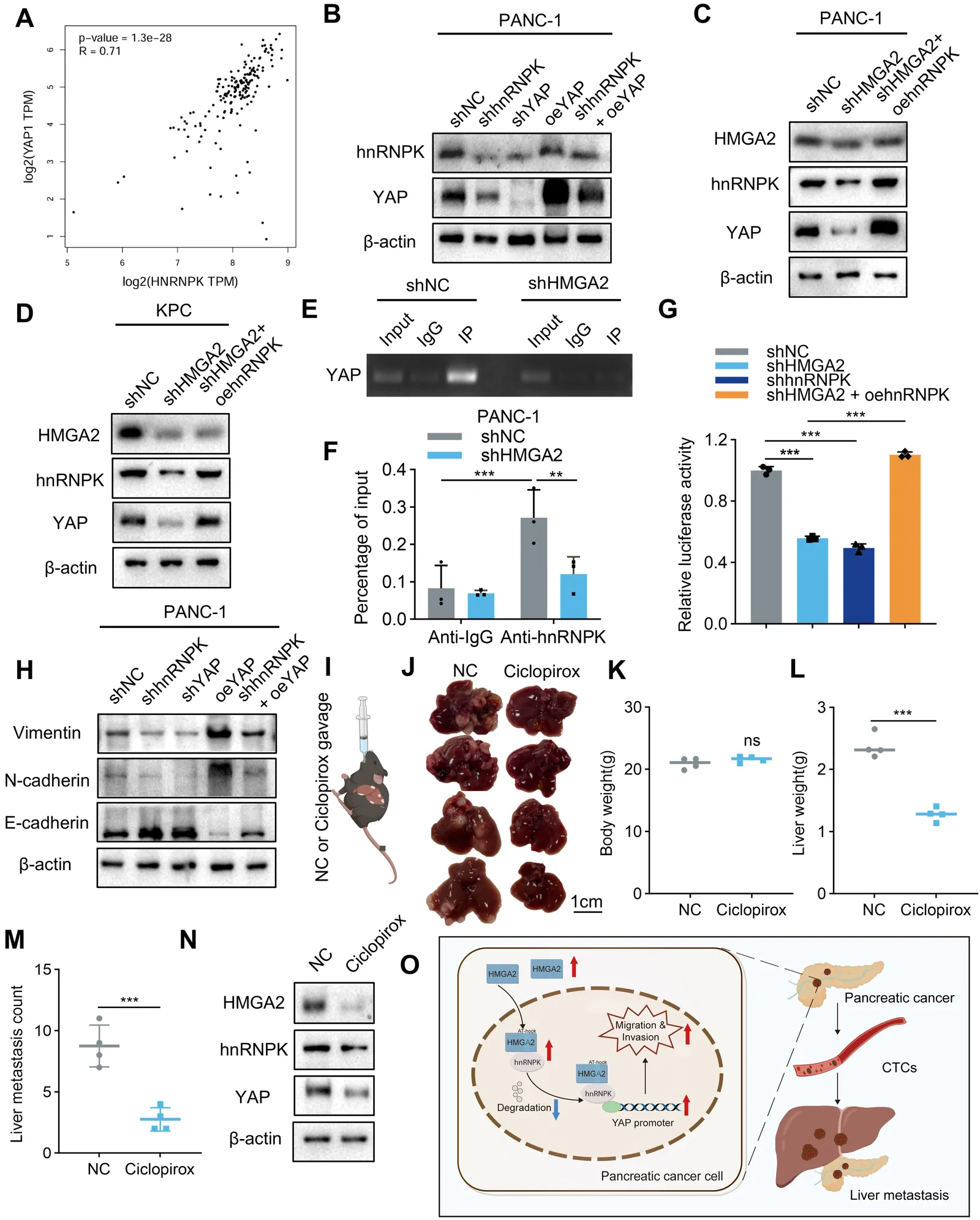
** hnRNPK binds to YAP promoter to activate its transcription. (A)** Spearman correlation analysis between hnRNPK and YAP in PC tissues. **(B)** Western blot of hnRNPK and YAP expression in shNC, shhnRNPK, shYAP, oeYAP and shhnRNPK + oeYAP PANC-1 cells. **(C)** Western blot of HMGA2, hnRNPK and YAP expression in shNC, shHMGA2, shHMGA2 + oehnRNPK PANC-1 cells. **(D)** Western blot of HMGA2, hnRNPK and YAP expression in shNC, shHMGA2, shHMGA2 + oehnRNPK KPC cells. **(E)** PCR results of YAP promoter in shNC and shHMGA2 PANC-1 cells. **(F)** qPCR results of YAP promoter in shNC and shHMGA2 PANC-1 cells (n = 3 independent experiments). **(G)** Luciferase reporter assay for YAP activity in the PANC-1 cells of shNC, shHMGA2, shhnRNPK and shHMGA2 + oehnRNPK group (n = 3 independent experiments). **(H)** Western blot of EMT markers expression in shNC, shhnRNPK, shYAP, oeYAP and shhnRNPK + oeYAP PANC-1 cells. **(I)** Experimental scheme of the gavage model: Six-week-old female C57BL/6 mice received intrasplenic injection of KPC cells and then were treated with NC or ciclopirox. **(J)** Liver images of NC and Ciclopirox groups after treatment. **(K-M)** Mouse body weight (K), liver weight (L) and liver metastatic nodules count (M) analysis of NC and Ciclopirox groups (n = 4 mice per group). **(N)** Western blot of HMGA2, hnRNPK and YAP expression in liver metastatic tissues from NC and ciclopirox-treated mice. **(O)** Schematic diagram showing that HMGA2 co-localizes with and binds to hnRNPK to interact therewith enabling hnRNPK to activate the YAP promoter for transcriptional induction ultimately facilitating LM of PC. Data are shown as mean ± SD. **P < 0.01, ***P < 0.001. Statistical significance was calculated using unpaired Student's t test (for 2 groups) or one-way ANOVA followed by Tukey's test (for more than 2 groups).
