# Supplementary material for: HMGA2 Stabilizes hnRNPK to Drive YAP-Dependent Liver Metastasis in Pancreatic Cancer
Source: Int J Biol Sci. 2026 Oct 2;22(15):8636–52. doi: 10.7150/ijbs.133139 (PMC13686062; doi:10.7150/ijbs.133139)
Supplement: Supplementary file 1 [file ijbsv22p8636s1.pdf]

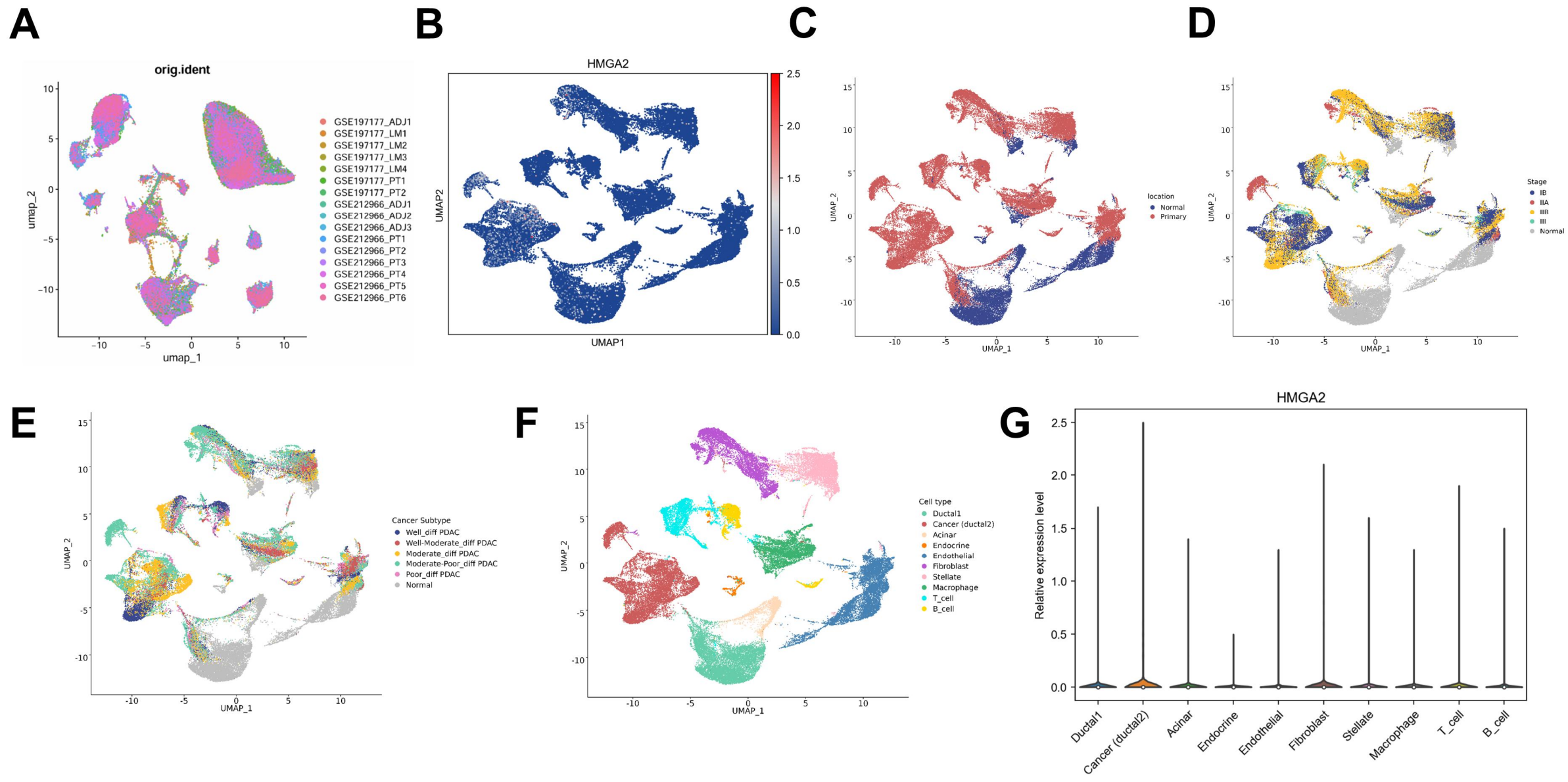

Figure S1. Bioinformatic analysis of HMGA2 expression in independent public single-cell RNA-seq datasets of human PC. (A) UMAP of the paratumor, primary tumor and LM tissue groups colored by sample identity. (B) UMAP of 24 PC and 11 normal pancreatic tissues colored according to HMGA2 expression. (C) UMAP split by normal and primary tumor tissue origin. (D) UMAP stratifying cells by clinical TNM stage. (E) UMAP grouping cells by differentiation cancer subtypes. (F) UMAP annotation of 10 distinct cell subtypes using lineage marker genes. (G) Violin plot quantitatively compares HMGA2 expression levels among different cell subsets.

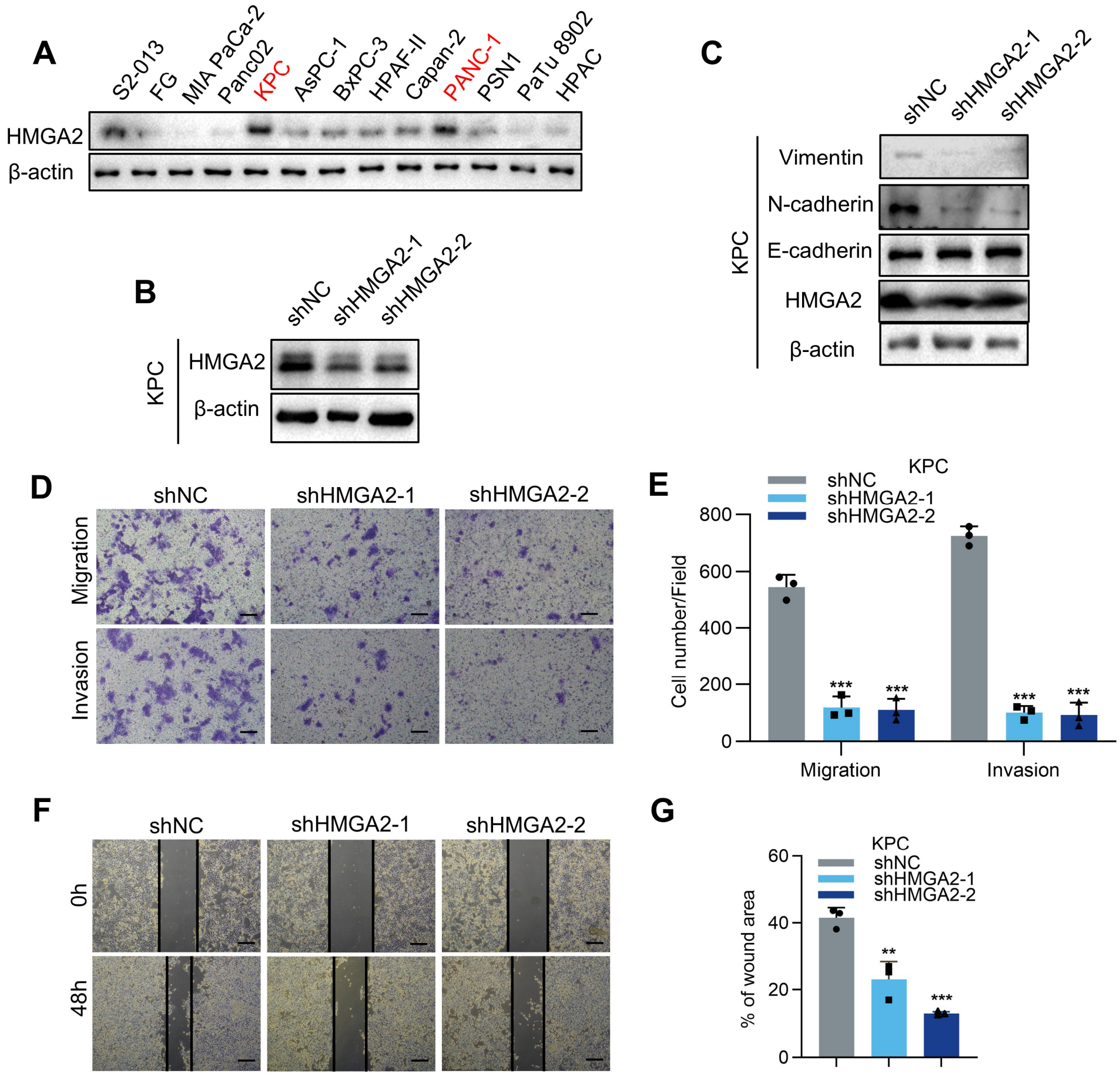

Figure S2. Knockdown of HMGA2 inhibits the migration and invasion ability of KPC cells. (A) Western blot of HMGA2 expression in multiple PC cell lines. (B) Western blot of HMGA2 expression in shNC, shHMGA2-1 and shHMGA2-2 KPC cells. (C) Western blot of EMT markers expression in shNC, shHMGA2-1 and shHMGA2-2 KPC cells. (D, E) Representative images of Transwell assays and quantification of the number for shNC, shHMGA2-1 and shHMGA2-2 KPC cells (n = 3 independent experiments). (F, G) Representative images of wound healing assay and quantification of the percentage of wound area for shNC, shHMGA2-1 and shHMGA2-2 KPC cells (n = 3 independent experiments). Data are shown as mean ± SD. ***P* < 0.01, ****P* < 0.001. Statistical significance was calculated using one-way ANOVA followed by Tukey's test (for 3 groups)

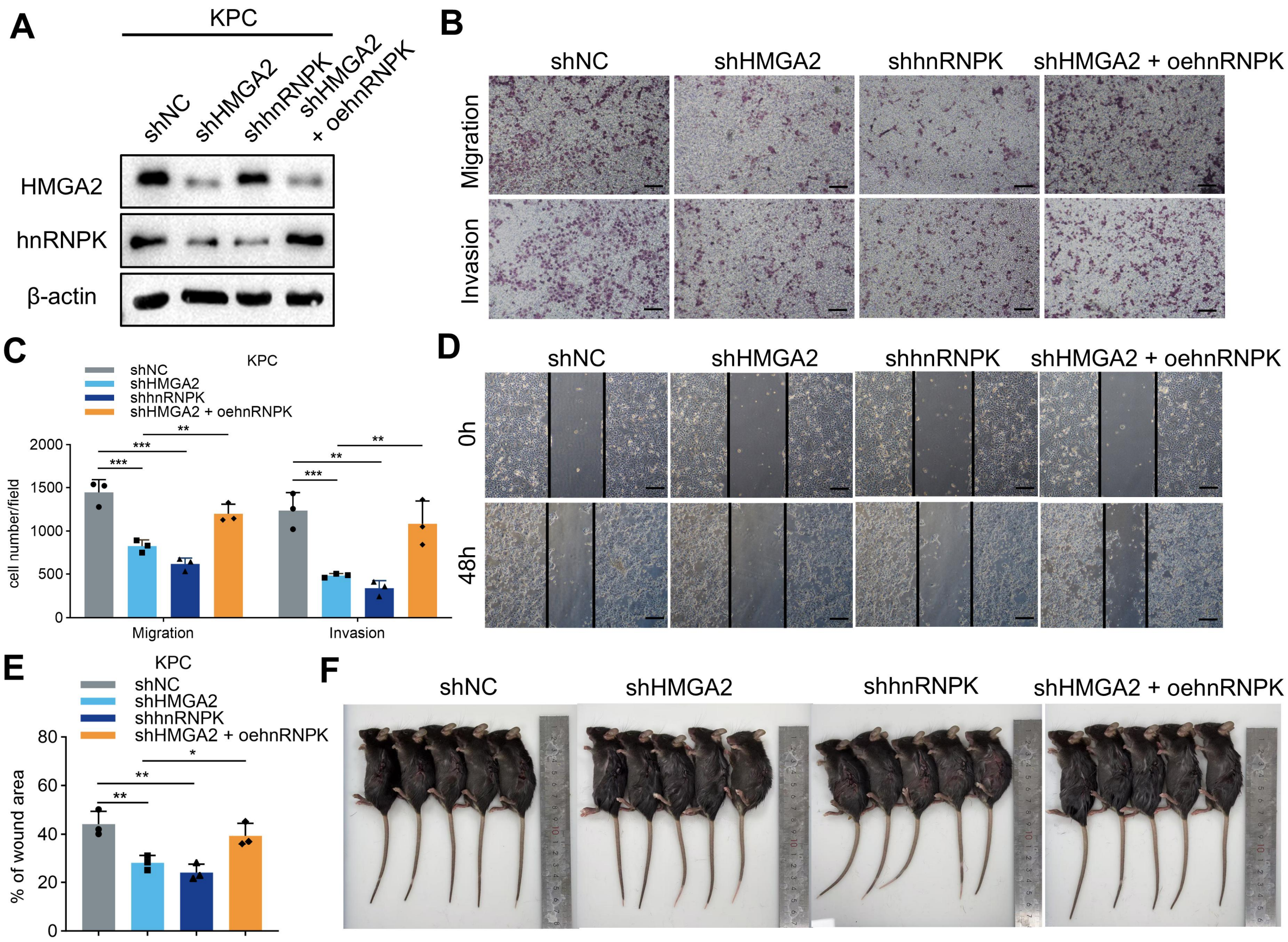

Figure S3. hnRNPK overexpression reverses the migration and invasion abilities inhibited by HMGA2 knockdown. (A) Western blot of HMGA2 and hnRNPK expression in shNC, shHMGA2, shhnRNPK and shHMGA2 + oehnRNPK KPC cells. (B, C) Representative images of Transwell assays and quantification of the number for KPC cells (n = 3 independent experiments). (D, E) Representative images of wound healing assay and quantification of the percentage of wound area for KPC cells (n = 3 independent experiments). (F) Mouse images of shNC, shHMGA2, shhnRNPK and shHMGA2 + oehnRNPK groups. Data are shown as mean ± SD. **P* < 0.05, ***P* < 0.01, ****P* < 0.001. Statistical significance was calculated using one-way ANOVA followed by Tukey's test (for 4 groups).

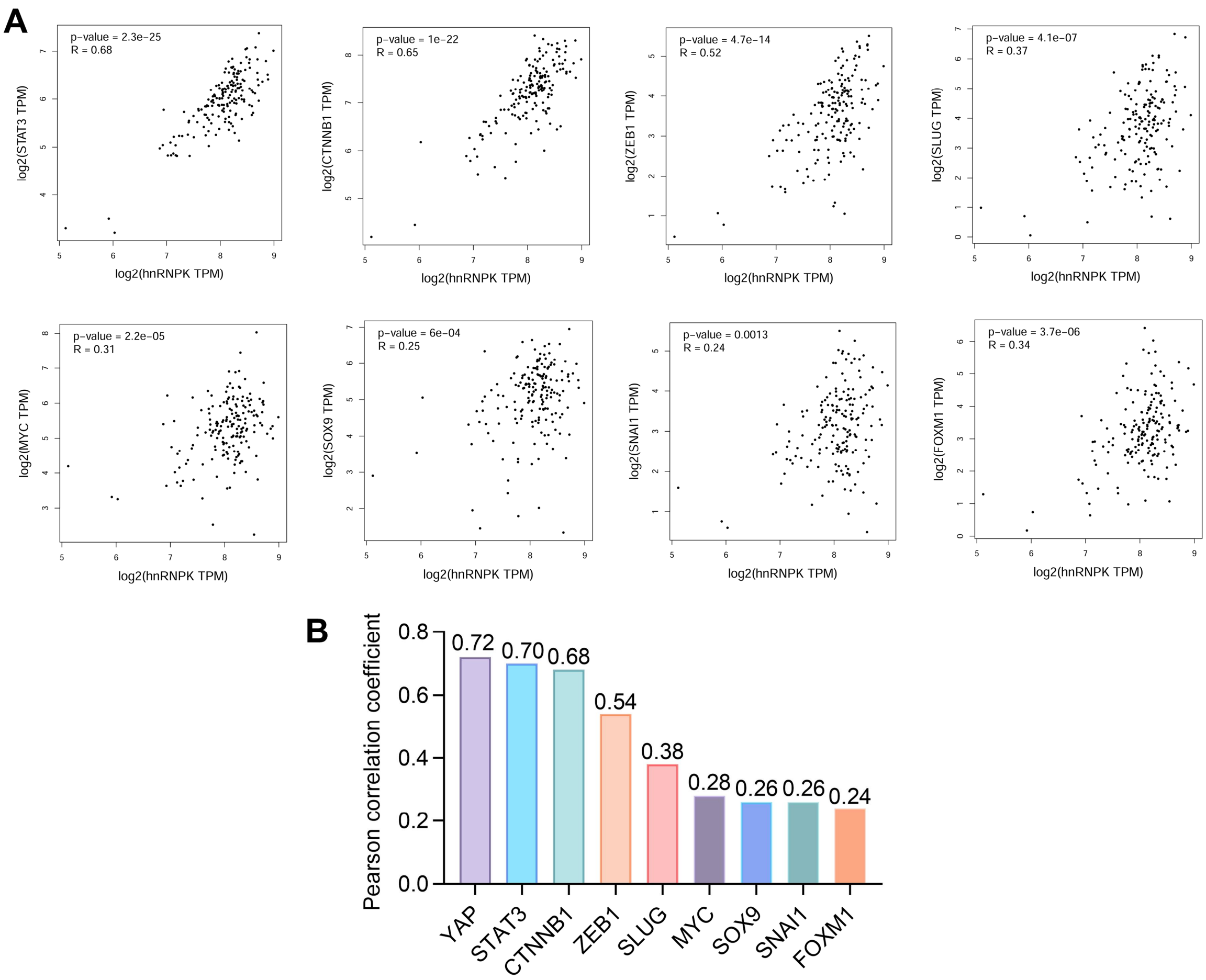

Figure S4. Correlation analysis between hnRNPK and its downstream transcriptional targets in PC. (A) Scatter plots displaying pairwise Spearman correlation for hnRNPK with downstream signaling mediators. (B) Quantification bar graph summarizing Pearson correlation coefficients of hnRNPK with downstream signaling mediators.

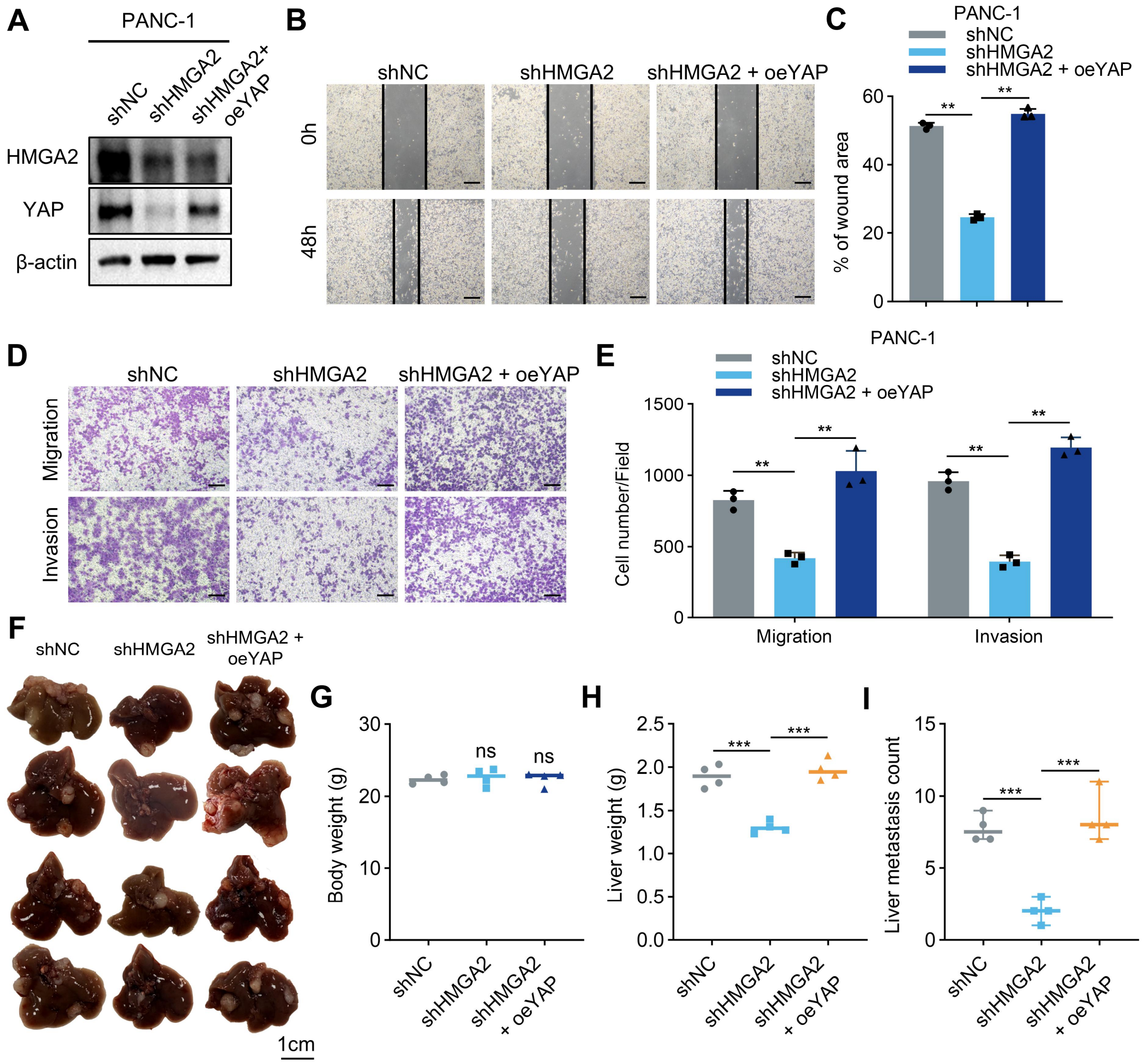

Figure S5. YAP overexpression reverses the inhibition of LM induced by HMGA2 knockdown. (A) Western blot of HMGA2 and YAP expression in shNC, shHMGA2 and shHMGA2 + oeYAP PANC-1 cells. (B, C) Representative images of wound healing assay and quantification of the percentage of wound area for PANC-1 cells (n = 3 independent experiments). (D, E) Representative images of Transwell assays and quantification of the number for PANC-1 cells (n = 3 independent experiments). (F) Liver images of shNC, shHMGA2 and shHMGA2 + oeYAP groups. (G-I) Mouse body weight (G), liver weight (H) and liver metastatic nodules count (I) analysis of shNC, shHMGA2 and shHMGA2 + oeYAP groups (n = 4 mice per group). Data are shown as mean ± SD. ***$P < 0.001$. Statistical significance was calculated using one-way ANOVA followed by Tukey's test (for 3 groups).

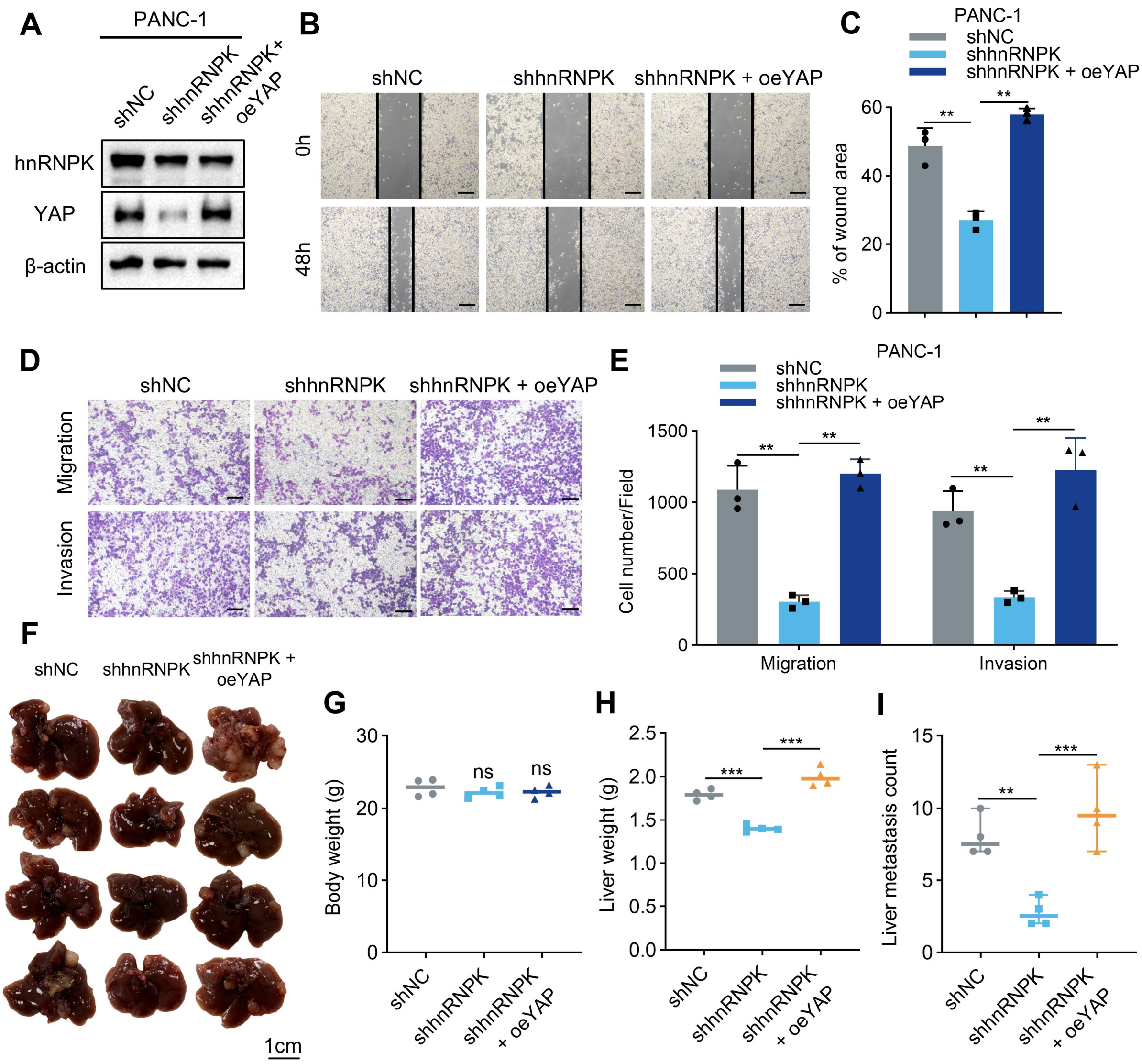

Figure S6. YAP overexpression reverses the inhibition of LM induced by hnRNPK knockdown. (A) Western blot of hnRNPK and YAP expression in shNC, shhnRNPK and shhnRNPK + oeYAP PANC-1 cells. (B, C) Representative images of wound healing assay and quantification of the percentage of wound area for PANC-1 cells (n = 3 independent experiments). (D, E) Representative images of Transwell assays and quantification of the number for PANC-1 cells (n = 3 independent experiments). (F) Liver images of shNC, shhnRNPK and shhnRNPK + oeYAP groups. (G-I) Mouse body weight (G), liver weight (H) and liver metastatic nodules count (I) analysis of shNC, shhnRNPK and shhnRNPK + oeYAP groups (n = 4 mice per group). Data are shown as mean ± SD. $^{**}P < 0.01$, $^{***}P < 0.001$. Statistical significance was calculated using one-way ANOVA followed by Tukey's test (for 3 groups).

Table S1. Clinicopathological characteristics of patients whose PC and LM tissues were used in RNA-seq

Variables	case #1	case #2	case #3	case #4
Gender	Male	Male	Male	Female
Age	57	65	63	54
Tumor location	Head	Head	Tail	Head
Tumor size (cm)	3.0	3.5	2.6	2.1
Tumor stage	IV	IV	IV	IV
Number of metastases	2	1	2	2

Table S2. Clinicopathological characteristics of patients whose PC and paratumor tissues were used in Western blot

Variables	case #1	case #2	case #3	case #4	case #5
Gender	Female	Male	Male	Female	Male
Age	56	72	59	53	67
Tumor location	Head	Tail	Tail	Head	Head
Tumor size (cm)	2.7	3.4	2.6	4.2	4.7
Tumor stage	II	II	III	III	II

Table S3. Clinicopathological characteristics of patients in LM and non-liver metastasis groups

Variables	case #1	case #2	case #3	case #4	case #5	case #6	case #7	case #8	case #9	case #10
Gender	Female	Male	Female	Female	Male	Male	Female	Female	Male	Male
Age	71	68	58	64	63	57	76	59	63	66
Tumor location	Tail	Head	Tail	Tail	Head	Head	Head	Head	Tail	Tail
Tumor size (cm)	3.6	2.5	3.8	4.2	3.9	4.8	3.7	4.4	3.6	4.2
Tumor stage	III	II	II	III	II	II	III	II	III	II
Time of postoperative LM (Month)	3	3	4	5	3	None	None	None	None	None

Table S4. The sequences of lentivirus and plasmids

Name	Sequences
shHMGA2-1	AGTCCCTCTAAAGCAGCTCAA
shHMGA2-1	AACGGCCAAGAGGCAGACCTA
shhnRNPK	GCCTCCATCTAGAAGAGATTA
shYAP	GCAGCAGAATATGATGAACTC
HMGA2 FL	ATGAGCGCACGCGGTGAGGGCGCGGGGCAGCCGTCCACTTCAGCCCAGGGACAACCT<br>GCCGCCCCAGCGCCTCAGAAGAGAGGACGCGGCCGCCCCAGGAAGCAGCAGCAAGA<br>ACCAACCGGTGAGCCCTCTCCTAAGAGACCCAGGGGAAGACCCAAAGGCAGCAAAAA<br>CAAGAGTCCCTCTAAAGCAGCTCAAAAGAAAGCAGAAGCCACTGGAGAAAAACGGCC<br>AAGAGGCAGACCTAGGAAATGGCCACAACAAGTTGTTCAGAAGAAGCCTGCTCAGGA<br>GGAAACTGAAGAGACATCCTCACAAGAGTCTGCCGAAGAGGACTAG
HMGA2 1-83	ATGAGCGCACGCGGTGAGGGCGCGGGGCAGCCGTCCACTTCAGCCCAGGGACAACCT<br>GCCGCCCCAGCGCCTCAGAAGAGAGGACGCGGCCGCCCCAGGAAGCAGCAGCAAGA<br>ACCAACCGGTGAGCCCTCTCCTAAGAGACCCAGGGGAAGACCCAAAGGCAGCAAAAA<br>CAAGAGTCCCTCTAAAGCAGCTCAAAAGAAAGCAGAAGCCACTGGAGAAAAACGGCC<br>AAGAGGCAGACCTAGGAAATGG
HMGA2 1-73	ATGAGCGCACGCGGTGAGGGCGCGGGGCAGCCGTCCACTTCAGCCCAGGGACAACCT<br>GCCGCCCCAGCGCCTCAGAAGAGAGGACGCGGCCGCCCCAGGAAGCAGCAGCAAGA<br>ACCAACCGGTGAGCCCTCTCCTAAGAGACCCAGGGGAAGACCCAAAGGCAGCAAAAA<br>CAAGAGTCCCTCTAAAGCAGCTCAAAAGAAAGCAGAAGCCACTGGAGAA
HMGA2 36-109	CAGCAAGAACCAACCGGTGAGCCCTCTCCTAAGAGACCCAGGGGAAGACCCAAAGGC<br>AGCAAAAACAAGAGTCCCTCTAAAGCAGCTCAAAAGAAAGCAGAAGCCACTGGAGAA<br>AAACGGCCAAGAGGCAGACCTAGGAAATGGCCACAACAAGTTGTTCAGAAGAAGCCT<br>GCTCAGGAGGAAACTGAAGAGACATCCTCACAAGAGTCTGCCGAAGAGGACTAG

Table S5. Clinicopathological characteristics of patients in High HMGA2 expression and Low HMGA2 expression groups

Variables		N	High HMGA2	Low HMGA2	*P* value
Gender					0.479
	Male	35	22	13	
	Female	26	14	12	
Age					0.804
	<60	28	17	11	
	≥60	33	19	14	
Differentiation					0.532
	Well	7	3	4	
	Moderately	16	8	8	
	Poorly	38	25	13	
T stage					0.478
	T1	7	3	4	
	T2	20	10	10	
	T3	25	15	10	
	T4	9	8	1	
N stage					0.648
	N0	47	27	20	
	N1	14	9	5	
M stage					**0.018**
	M0	50	26	24	
	M1	11	10	1	
Classification of TNM					**0.013**
	I-II	43	21	22	
	III-IV	18	15	3	

Table S6. The mass spectrometry screening of HMGA2 immunoprecipitation complexes detected multiple hnRNP family proteins

Acc.	Gene	Ctrl		Probe		Protein Name
		Num Unique	Peptide Count	Num Unique	Peptide Count	
P61978	hnRNPK	0	0	31	117	Heterogeneous nuclear ribonucleoprotein K
P55795	hnRNPH2	0	0	4	6	Heterogeneous nuclear ribonucleoprotein H2
O43390	hnRNPR	0	0	2	3	Heterogeneous nuclear ribonucleoprotein R
Q14103	hnRNPD	0	0	2	3	Heterogeneous nuclear ribonucleoprotein D
